# Supplementary material for: ‘Treading water but drowning slowly’: what are GPs’ experiences of living and working with mental illness and distress in England? A qualitative study
Source: BMJ Open. 2018 May 3;8(5):e018620. doi: 10.1136/bmjopen-2017-018620 (PMC5942433; doi:10.1136/bmjopen-2017-018620)
Supplement: Author's manuscript [file bmjopen-2017-018620.draft_revisions.pdf]

BMJ Open

BMJ Open is committed to open peer review. As part of this commitment we make the peer review history of every article we publish publicly available.

When an article is published we post the peer reviewers' comments and the authors' responses online. We also post the versions of the paper that were used during peer review. These are the versions that the peer review comments apply to.

The versions of the paper that follow are the versions that were submitted during the peer review process. They are not the versions of record or the final published versions. They should not be cited or distributed as the published version of this manuscript.

BMJ Open is an open access journal and the full, final, typeset and author-corrected version of record of the manuscript is available on our site with no access controls, subscription charges or pay-per-view fees (http://bmjopen.bmj.com).

If you have any questions on BMJ Open's open peer review process please email editorial.bmjopen@bmj.com

BMJ Open

‘Treading water but drowning slowly’: What are GPs’ experiences of living and working with mental illness and distress in England – a qualitative study

Journal:	*BMJ Open*
Manuscript ID	bmjopen-2017-018620
Article Type:	Research
Date Submitted by the Author:	10-Jul-2017
Complete List of Authors:	Riley, Ruth; University of Birmingham, of Applied Health Research; University of Bristol, Social and Community Medicine<br>Spiers, Johanna ; University of Bristol School of Social and Community Medicine<br>Chew-Graham, Carolyn; University of Keele, Primary Care and Health Sciences<br>Taylor, Anna ; University of Bristol, Social and Community Medicine<br>Thornton, Gail; University of Bristol, Social and Community Medicine<br>Buszewicz, Marta; University College London, Research Department of Primary Care and Population Health
**Primary Subject Heading**:	General practice / Family practice
Secondary Subject Heading:	Mental health, Qualitative research, Occupational and environmental medicine
Keywords:	General Practitioners, Depression, Anxiety, Distress, Chronic Stress, Suicidal Ideation

SCHOLARONE™
Manuscripts

‘Treading water but drowning slowly’: What are GPs’ experiences of living and working with mental illness and distress in England – a qualitative study

Ruth Riley

Lecturer

Institute of Applied Health Research

University of Birmingham
Birmingham

United Kingdom

r.riley@bham.ac.uk

0121

Johanna Spiers

Senior research associate

Centre for Academic Primary Care

University of Bristol

United Kingdom

0117 33 14532

Js16447@bristol.ac.uk

Carolyn A Chew-Graham

Professor of General Practice Research

Research Institute

Primary Care and Health Sciences

Keele University

Keele

United Kingdom

01782 734 717

c.a.chew-graham@keele.ac.uk

Anna Kathryn Taylor

Medical Student,

For peer review only - http://bmjopen.bmj.com/site/about/guidelines.xhtml

Faculty of Health Sciences,
University of Bristol
Bristol
United Kingdom
0117 331 6825
at0012@my.bristol.ac.uk

Gail Thornton
Patient and Public contributor
Centre for Academic Primary Care
University of Bristol
United Kingdom
0117 9104917
gail@thorntonuk.com

Marta Buszewicz
Reader in Primary Care
Research department of Primary Care and Population Health
University College London
London
United Kingdom
020 7794 0500 extn. 31016
m.buszewicz@ucl.ac.uk

Word count: 3,596

Abstract

Objectives. This paper reports General Practitioners' (GPs) experiences of living and working with mental illness and distress as part of a wider study exploring the barriers and facilitators to help-seeking for mental illness and burnout amongst this medical population.

For peer review only - http://bmjopen.bmj.com/site/about/guidelines.xhtml

Design. Qualitative study using in-depth interviews with 47 GP participants. The interviews were audio-recorded, transcribed, anonymised and imported into NVivo11 to facilitate data management. Data were analysed using a thematic analysis employing the constant comparative method.

Setting. England

Participants. A purposive sample of GPs participants who self-identified as: 1) currently living with mental distress, 2) returning to work following treatment, 3) off sick or retired early as a result of mental distress or 4) without experience of mental distress. Interviews were conducted face to face or over the telephone.

Results. GP participants reported living and working with poor mental health, with many recollecting their distressing experiences and significant psychological and physical symptoms relating to chronic stress, anxiety, depression and/or burnout, and almost half articulating thoughts of suicide. Many talked about their shame, humiliation and embarrassment at their perceived inability to cope with the stresses of their job and/or their symptoms of mental illness.

Conclusions. These findings paint a concerning picture of the situation affecting primary care doctors, with participants' accounts suggesting there is a considerable degree of mental ill health and reduced wellbeing amongst GPs. The solutions are complex and lie in prevention and provision. There needs to be greater recognition of the components and cumulative effect of occupational stressors for doctors, such as the increasing workload and the clinical and emotional demands of the job, as well as the need for a culture shift within medicine to more supportive and compassionate work environments.

Keywords: Qualitative, General Practitioners, Depression, Anxiety, Distress

Strengths and limitations of this study

- Few studies, employing qualitative methods, using in-depth interviews have been utilised to examine this topic – this study carried out 47 interviews with GPs from across England and contributes to a growing body of research illuminating and examining the causes and impact of chronic stress and distress amongst GPs

- Participants were self-selecting, which may be perceived as a limitation; however, the sampling of participants ensured that the sample was varied in terms of age, gender, number of years as practising GPs, level of seniority/employment status in the practice, and geographical location

- Due to time constraints, the researchers were unable to employ respondent validation; however, the second coders included academic GPs and team members with lived experience of mental illness which afforded some checks and balances to

For peer review only - http://bmjopen.bmj.com/site/about/guidelines.xhtml

the validity of the analytic process, interpretation of data and generalizability of the research findings.

- In recruiting individuals with experience of mental illness and burnout, we also included the perspectives of GPs who had no disclosed history of mental illness, which enables the data to be more widely generalizable to the GP population

- The multi-disciplinary research team independently analysed a sub-set of transcripts in order to contribute to the generation and refinement of codes to maximise rigour; emergent themes were subsequently discussed by the whole team to ensure credibility and confirmability.

INTRODUCTION

Compared with the general population, doctors, including general practitioners (GPs) working in primary care, experience high levels of psychological distress linked to chronic stress, anxiety, depression and burnout [1-4]. These doctors also have higher rates of mental illness and suicide [5-8]. Evidence from industrialized countries (e.g. UK, USA, Australia), indicates that suicide rates are higher amongst GPs, psychiatrists and anaesthetists compared with other medical specialities [6 8]. Doctors undergoing fitness for practice investigations are at particularly high risk of suicide [9]. Compared with the general population, doctors also have higher rates of drug and alcohol misuse and dependency, with a higher rate of misuse of prescription medicines [10]. Psychological difficulties or distress experienced by doctors can be further compounded by self and societal stigma associated with mental health illness, with evidence that they avoid or delay disclosure and help-seeking [11 12]. The prevailing hegemonic culture amongst medical practitioners and non-disclosure of invulnerability, with shame attached to mental health problems or psychological difficulties and the consequent perceived inability to cope within the job, also hinders help-seeking [4 11 13 14]. It has been suggested that personality characteristics such as perfectionism and obsessionality whilst being positively associated with hard-working and conscientious professionals, may play a role in predisposing doctors towards mental ill-health [4].

The increasing prevalence of mental ill-health amongst the medical profession, including GPs, has been attributed to a number of factors, most notably the escalating occupational stresses related to workload, long hours and the emotional demands of the job [4 15-19]. Evidence from UK data indicates that GPs face an increase in patient demand, evidenced by a 16% increase in workload over the past seven years [16], as well as workforce shortages and a reduction in resources [13 15 20]. There has been an increase in the transfer of care from specialist to primary care without a commensurate increase in resources which has also added to the pressures in this setting. Currently, 90% of patient consultations take place in

For peer review only - http://bmjopen.bmj.com/site/about/guidelines.xhtml

general practice, yet the allocated budget accounts for only 8% of the NHS total [21 22]. Consequently, the pressure on primary care in England is currently at its highest ever, and is predicted to increase in the future [22].

Evidence of the extent of mental ill-health and burnout amongst GPs is largely derived from survey data, with some small-scale qualitative studies exploring the topic [23-27]. To date, few studies have explored the experiences and accounts of GPs living and working with enduring levels of stress, mental ill-health and psychological distress using in-depth qualitative methods. This paper reports GPs' experiences of living and working with mental illness and emotional distress, as part of a wider study exploring the sources of stress and distress for GPs and the barriers and facilitators to help-seeking for mental illness and burnout amongst this medical population.

METHODS

This multi-centre, qualitative study employed in-depth interviews with 47 GPs in England. Information about the study was advertised through professional publications such as Pulse, study newsletters, social media and national and local GP networks (i.e. Local Medical Committees or LMCs) in Bristol, Manchester and London. A sub-sample (n=12) was recruited through a specialist treatment service. GPs interested in taking part were asked to self-select into the following groups: 1: Living with anxiety, depression, stress and/or, burnout; 2: Returning to work following treatment; 3: Off sick or retired early due to psychological ill-health 4: No mental ill-health. GPs were reimbursed £80 to recognise the time for their participation.

Participating GPs were reimbursed with £80 to recognise the time for their participation. Interested GPs were purposively sampled to represent as even a spread as possible across these four groups, although the largest number of participants were in group 1. We intended to purposively sample approximately 10 participants per group. However, the majority of GPs who contacted us self-selected into group one, and due to the emergent rich data continued recruitment to this group and further exploration of emerging themes was justified in meeting the study's aims and objectives. We endeavored to recruit more participants into groups two and four using targeted publicity information, but because of time constraints those groups remained marginally under-recruited. In the event, many GPs who identified as living with no stress reported as having had experiences of stress and distress at some juncture in their career. More female GPs contacted the study team expressing an interest in participating, and therefore the disparity in numbers reflects this. The iterative process of recruitment, sampling and analysis ensured that emerging concepts and themes could be tested out amongst participants with different characteristics (e.g. partners vs locums GPs).

Face-to-face or telephone interviews lasting between 27-126 minutes (mean = 69 minutes) were conducted between April and September 2016. The majority of interviews were conducted in participants' homes. The in-depth interviews were conducted by two authors

For peer review only - http://bmjopen.bmj.com/site/about/guidelines.xhtml

(JS, RR) using a flexible topic guide (see Box 1). This was informed by the existing literature, input from GPs on the study team and PPI consultation exercises conducted with GP networks prior to obtaining funding. The interviews were audio-recorded, transcribed, anonymised and imported into NVivo11 to facilitate data management. Analysis and data collection were conducted iteratively. A thematic analysis was performed involving a process of constant comparison between cases [28]. Analysis commenced with JS generating an initial coding framework grounded in the data, which was added to and refined, with material regrouped and recoded as new data were gathered. Codes were gradually built into broader categories through comparison across transcripts, and higher-level recurring themes were developed until data saturation was reached and no new themes were arising from the data[29]. Reflexivity was employed throughout the research. Both interviewers were experienced qualitative researchers who both reflected on and discussed the impact of the data on their cognitive and emotional sensing throughout the study. Both researchers also discussed and made explicit how their epistemological (JS with a background in psychology and RR in medical sociology) and experiential backgrounds may have oriented the data collection and analytic process.

The multi-disciplinary research team independently analysed a sub-set of transcripts in order to contribute to the generation and refinement of codes to maximise rigour. Emergent themes were discussed by the whole team to ensure credibility and confirmability.

RESULTS

Willing participants were purposively sampled from GPs who expressed an interest in the study based on demographic and practice characteristics as well as self-selected group type. The characteristics of the participants are found in Table 1.

<<<<insert table 1 here>>>>>

Analysis of the interview transcripts and field notes identified five main themes and corresponding sub themes relating to:

1. Burnout - loss of empathy or compassion fatigue, feeling empty and/or exhausted, difficulty making decisions, low job satisfaction and loss of enjoyment in seeing patients
2. Anxiety - psychological symptoms relating to anxiety, including feeling anxious or on edge, worrying, irritability, inability to relax and physical symptoms related to anxiety such as panic attacks, hyperventilating, palpitations, sweating, and nausea
3. Depression - distress, insomnia and exhaustion, negative thoughts, low self-esteem irritability and anger
4. Suicidal ideation - thoughts of suicide
5. Shame and feelings of failure and humiliation, resulting from a perceived lack of resilience and inability to cope

For peer review only - http://bmjopen.bmj.com/site/about/guidelines.xhtml

It should be noted that some participants described or disclosed symptoms that could be clearly attributed to chronic stress, anxiety, depression and burnout. Other participants described or framed their symptoms in terms of diagnostic concepts associated with these categories, whilst others talked about their feelings and emotional state without necessarily labelling them. Notably, the symptomology was identified across all groups including Group 4, that is, individuals who self-identified as having no mental health concerns. The themes also applied to participants irrespective of seniority or role in the practice, age or gender.

1. Symptoms of Burnout

Some participants reported feeling a loss of empathy or compassion fatigue, feeling empty, exhaustion, difficulty making decisions, low job satisfaction and loss of enjoyment for their work:

I'd lost my empathy with my patients. You know, and er I'm just – I was just – you're just like a ketchup bottle in a production line […] But from a personal point of view I'm still feeling a bit washed out by it, you know, I feel a little bit empty. You know, I feel like I'm burnt out really a bit, to be honest. (P25, Male Salaried)

I was saying all the right things but […] I felt quite detached from it. (P27, Female Salaried)

The following participant describes the impact on feeling overworked and their reduced ability to offer compassion to their patients:

If you find yourself working too many sessions, I can, you know, I feel it, and you almost feel like you lose the milk of human kindness. And you can't do that job if you're not (pause) at your best. (P21, Female, Salaried and Locum)

2. Symptoms of Anxiety

The majority of participants, including those who self-identified as having no mental health concerns (Group 4), described psychological symptoms relating to anxiety, including feeling on edge or hyper-alert, persistent worrying, irritability and an inability to relax.

Everything was running around in my mind at 200 miles an hour. I was in sort of hyper drive and, yeah, you wake up to super stimulated, super awake, super, you know, on edge and anxious. (P36, Male Partner)

I remember once actually sitting under the desk, hugging my knees to my chest, I was so anxious. (P25, Male Salaried)

Many participants also described physical symptoms related to their anxiety, such as panic attacks, hyperventilating, palpitations, sweating, and nausea:

Erm (pause) and I, and I was getting quite bad chest pain, palpitations, I was feeling anxious in many circumstances, like the school run, just meeting people, going to meetings and it

For peer review only - http://bmjopen.bmj.com/site/about/guidelines.xhtml

was becoming incapacitating where it felt like somebody had got a knife in my chest all the time. And I knew it wasn't anything to do with my heart. (P31, Female Locum)

3. **Symptoms of Depression**

Many GP participants described symptoms relating to depression, which included: distress, low mood, hopelessness, sleep problems, low self-esteem, guilt, restlessness, fatigue and suicidal feelings. Some participants employed a range of poignant and powerful metaphors to talk about their emotional state:

And, to be honest with you, that kind of er – the best way that I could describe that is that it's kind of like a dark shadow that's in the corner of the room […] I basically felt like I was treading water but drowning slowly. (P45, Male Salaried)

Distress:

Many participants recalled their experiences of feeling distressed and tearful, with some describing breaking down at home, after or during work or in front of patients:

I mean there were times certainly when I arrived at work and just sat outside and cried before I even went in the building. And I often cried on the way home. I'd often cry when I came in from work, um just collapse in a heap, and get to bed actually crying (P31, Female Locum)

I would just end up in tears all the time at the end of each session. (P53, Female Salaried

Insomnia and exhaustion:

Amongst the myriad of symptoms, insomnia and exhaustion were a commonplace experience for study participants:

Um - so I wasn't sleeping and I wasn't - wasn't eating very well, and - and it just all sort of took its toll. (P23, Female Salaried)

It's very hard to function when you're not ever getting refreshing sleep […] And I just wouldn't stop. And absolute, incapacitating terror. Um - So, but one's having um — night, night terrors maybe two, three, four times a week. Sometimes more than one a night. (P24, Female Partner)

Many GP participants detailed their exhaustion which was often linked to insomnia and could be symptomatic of their anxiety, depression and/or feeling burnt out.

Probably like sort of swimming through treacle, so everything seemed - um - much harder. (P38, Male Partner)

For peer review only - http://bmjopen.bmj.com/site/about/guidelines.xhtml

So like you can't, you can't avoid that fatigue. You might be able to put it off a bit (laughs) but you're going to pay interest on it. (P7, Female Locum)

Negative thoughts/low self-esteem:

Some participants talked about grappling with cycles of negative thoughts, symptomatic of low self-esteem and a common symptom of depression, as this participant illustrates:

And so then I'm really struggling, because I'm struggling with my internal dialogue saying, you know, "Not good enough, could be doing a better job. Not really, not really er making people better." […] persistent negative cycles of er (pause), yeah, of negative thoughts about yourself (P20, Male Partner)

Irritability and anger:

Some study participants described feeling irritable and sometimes angry; a possible symptom of depression in this context:

But I think, towards the end when I stopped locum-ing again, I was beginning to feel very tired as a result and that it was taking more out of me than it ought to really, and feeling um, yeah, feeling more irritable. (P47, Female on sick leave)

…it was fairly um traumatic really, I have to say…. I think I'd become quite stressed at work actually and I'd lost my temper in meetings at times and um nobody quite knew what to do about it. (P39, Male Partner)

4. Suicidal ideation

Almost half of the GP participants described having had suicidal thoughts, with the following participants reflecting on occasions when they had contemplated and ruminated on ways of taking their own lives:

And it just came on me like a wave. And then I started thinking, "I'm going to jump onto those train tracks." (P45, Male Salaried)

And um constant ruminations on suicide and doing myself in. And the trick with suicide is to persuade yourself that those around you would be better off without you, um and I got to that point and I decided on ways that I might (pause) do myself in and ruminated about that all the time. Just, it just filled my waking and sleeping nights. (P20, Male Partner)

The following GP reflects on the advice she gives to her patients who express suicidal thoughts and how her feelings mirrored her patients' expressed hopelessness and despair:

Um, and then I think the hard thing, the worst thing I think I found was when people were feeling suicidal, and they were saying they were feeling suicidal and why they were feeling suicidal. And normally I'd be like, "Look, you know, it's temporary, it'll get better. These are

For peer review only - http://bmjopen.bmj.com/site/about/guidelines.xhtml

the things that we can do," but I just sat there thinking, "Well, you're probably right, it is rubbish," you know. (P27, Female Partner)

5. Shame and feelings of failure

Perhaps symptomatic of the stigma attached to mental illness and culture of invulnerability within medicine, some GP participants described how they felt ashamed, embarrassed, humiliated and a sense of having failed, due to their perceived lack of resilience and inability to cope:

And you do feel a huge sense of shame, huge sense of shame. Because even though I treat patients day in, day out with anxiety and depression, it felt like this was my not coping. And I was thinking, "Why are the partners all able to cope with this but not me?" and that sort of thing. So it was all rolled into one really. (P4, Female Partner)

Er (sigh) yeah, I think for me it was very embarrassing. Didn't want to admit it. Couldn't believe that like I'd got to this stage. Cos I'd never been someone like that […] yeah, I did find it really hard, I think, to actually accept that this had happened. Um (pause) yeah, bit of a failure? I suppose, in a way. Kind of feel like you're not strong enough? (P44, Female Registrar)

DISCUSSION

The accounts of the 47 participants in this study suggest there may be a significant population of GPs who are living and working with poor mental health. Participants vividly described and recollected their distressing experiences and troubling symptoms, with almost half of the sample articulating thoughts of suicide. The GP participants described numerous significant psychological and physical symptoms relating to chronic stress, anxiety, depression and/or burnout. Many reported extreme levels of distress, with several breaking down at work – crying, feeling despair and some contemplating suicide. Many also talked about their shame, humiliation and embarrassment at their perceived inability to cope with the stresses of their job and/or their symptoms of mental ill-health. The experiences and feelings of many GP participants depict a dispiriting emotional landscape, with notable signs of poor mental health and striking levels of distress.

There has been a paucity of in-depth qualitative research exploring this topic area with doctors, including GPs. This study highlights that many GPs are living *and working* with extreme levels of emotional distress. A notable proportion of participants vividly recalled and articulated both fleeting and recurring thoughts of suicide, which is reflective of the extent of individuals' distress and suffering. These candid, evocative and sometimes disturbing accounts, some hitherto unvoiced, reveal a population of doctors with generally very poor mental health. They suggest that many GPs are living and working with enduring and distressing symptoms associated with anxiety, depression and/or burnout, yet still feel considerable shame and embarrassment associated with the perception that they are not coping. Some participants had taken time off work with severe symptoms and consequently

For peer review only - http://bmjopen.bmj.com/site/about/guidelines.xhtml

sought help, but many experienced barriers to effective help-seeking and treatment (reported in a separate paper).

The findings from this study were based on in-depth interviews with 47 GPs from across England and contribute to a growing corpus of research illuminating and examining the causes and impact of chronic stress and distress amongst GPs (Chesire et al. 2017, Doran et al. 2016a, Fisher et al. 2017. The participants were self-selecting, which may be seen as a limitation; however, the sampling of participants ensured that the sample was varied in terms of age, gender, number of years as practising GPs, level of seniority/employment status in the practice, and geographical location. In addition to recruiting individuals with self-reported mental ill-health and burnout, we also included the perspectives of GPs who had no disclosed history of mental health problems, which enables the data to be more widely generalizable to the GP population.

These findings present a concerning picture of psychological difficulties affecting primary care doctors, with participants reporting a considerable number of mental health problems and a significant degree of reduced wellbeing. The experience of chronic stress and anxiety in GPs (e.g. feeling tense, restless, anxious, worried or having disrupted sleep, panic attacks, hyperventilating, palpitations, sweating, and nausea) appears commonplace, including in those who did not identify themselves as having mental ill-health. There is also evidence that doctors frequently normalise, minimise, deflect or downplay how stressed they feel, which may preclude help-seeking and the availability of appropriate support [30]. Evidence indicates that chronic stress negatively impacts on physical health and is a key risk factor for mental illness, including anxiety and depression, and can lead to the adoption of maladaptive coping mechanisms such as nicotine, alcohol, and illicit drug use [31].

Normalising and acknowledging chronic stress as an occupational hazard associated with being a doctor is helpful, but only if solutions are sought which focus on prevention and the provision of support to maintain the health and wellbeing of frontline clinicians. Since January 2017, GPs and GP trainees in England suffering from mental ill health and addiction have been able to access free support through the National Health Service's GP Health Service [32]. Further research and evaluation must be undertaken to assess the effectiveness of this service. The emphasis now needs to focus on preventing mental ill health and improving the support available to doctors. Crucially, collegial support is a protective factor for good mental health - support from mentors, supervisors and colleagues is associated with resilience and reduced sickness [19]. Balint groups or similarly structured group work or supervision continue to be employed in general practice and are valued by GPs [33], yet are not used across all practices. Individual or group supervision aims to provide a safe and supportive space where staff can openly discuss the pressures and emotional challenges of their work and may, as previous evidence suggests, provide GPs with the support they need whilst offering protection against compassion fatigue and burnout [34]. Its wider use may need to be considered.

Tackling the culture of invulnerability early on in medical training is also key. Schwartz Centre Rounds®, for instance, are currently being piloted in medical schools with early

For peer review only - http://bmjopen.bmj.com/site/about/guidelines.xhtml

evidence supporting their value [35]. First5®, buddy systems, and access to regular supervision or mentorship can also offer support and a reflective space. The British Medical Association (BMA) have produced guidance on recognising signs of mental distress amongst colleagues with suggestions about what action to take [36]. Perhaps caution needs to be applied to the over-reliance on individual resilience as a solution as this may shift the responsibility for maintaining good mental health with the person. While self-care is paramount in maintaining good mental and physical health, it is not solely the responsibility of doctors to look after their mental health; employers and colleagues need to engender a more supportive and compassionate culture. Doctors are not invulnerable to suffering, most especially their own.

In the light of the findings of this study, we recommend the following:

- Greater recognition of the components and cumulative effect of the occupational stressors for doctors, such as increasing workload and the clinical and emotional demands of the job
- A culture change within medicine, with a shift to more supportive and compassionate work cultures
- The de-stigmatising of mental ill-health and the recognition of GPs' experiences and emotional responses as symptomatic of occupational stress

Conclusion

These findings paint a concerning picture of the situation affecting primary care doctors, with participants' accounts suggesting there is a considerable degree of mental ill health and reduced wellbeing amongst GPs. The solutions are complex and lie in prevention and provision. There needs to be greater recognition of the components and cumulative effect of occupational stressors for doctors, such as the increasing workload and the clinical and emotional demands of the job, as well as the need for a culture shift within medicine to more supportive and compassionate work environments.

Author Contribution:

RR, JS, CCG, MB– substantial contributions to conception and design, acquisition of data, or analysis and interpretation of data; drafting the article or revising it critically for important intellectual content; and final approval of the version to be published.

AT & GT - analysis and interpretation of data; drafting the article or revising it critically for important intellectual content; and final approval of the version to be published.

Acknowledgements: The authors would like to thank all volunteer participants who kindly gave their time to share their experiences of working in English general practice; and collaborators Dr. Clare Gerada and Dr. Chris Manning for endorsing this study and who provided valuable comments on the funding application and who assisted with recruitment.

Competing interests: None

For peer review only - http://bmjopen.bmj.com/site/about/guidelines.xhtml

Funding: The study was funded by NIHR School for Primary Care Research

Department of Health Disclaimer: The views and opinions expressed therein are those of the authors and do not necessarily reflect those of the NIHR School for Primary Care Research, NIHR, NHS or the Department of Health.

Ethical approval: Ethical approval was granted by the South West - Frenchay Research Ethics Committee (reference number: 15/SW/0350)

Data sharing statement: This study has not received ethical approval to share confidential data with any third party other than the study research team.

For peer review only

REFERENCES

1. Chopra SS, Sotile WM, Sotile MO. Physician burnout. JAMA 2004;**291**(5):633-33.
2. Merlo LJ, Singhakant S, Cummings S.M, Cottler L.B. Reasons for Misuse of Prescription Medication Among Physicians Undergoing Monitoring by a Physician Health Program. Journal of addiction medicine 2013;**7**(5):349-53.
3. Orton P, Orton C, Pereira Gray D. Depersonalised doctors: a cross-sectional study of 564 doctors, 760 consultations and 1876 patient reports in UK general practice. BMJ Open 2012;**2**(1).
4. Brooks SK, Gerada C, Chalder M. Review of literature on the mental health of doctors: Are specialist services needed? Journal of Mental Health 2011:1-11.
5. Dobbin A. Burnt out or fired up? Helping recovery in distressed patients can increase our own resilience. Br J Gen Pract 2014;**64**(627):497-98.
6. Hawton K, Clements A, Sakarovitch C, et al. Suicide in doctors: a study of risk according to gender, seniority and specialty in medical practitioners in England and Wales, 1979–1995. Journal of Epidemiology and Community Health 2001;**55**(5):296-300.
7. Richings JC, Khara GS, Mcdowell M. Suicide in young doctors. . British Journal of Psychiatry 1986;**149**:475-78.
8. Schernhammer ES, Colditz GA. Suicide Rates Among Physicians: A Quantitative and Gender Assessment (Meta-Analysis). American Journal of Psychiatry 2004;**161**(12):2295-302.
9. GMC. Doctors who commit suicide while under GMC fitness to practise investigation. 2014.
10. Brooks SK, Chalder T, Gerada C. Doctors vulnerable to psychological distress and addictions: treatment from the Practitioner Health Programme. . J Ment Health 2011;**20**:157–64.
11. Henderson M, Brooks SK, del Busso L, et al. Shame! Self-stigmatisation as an obstacle to sick doctors returning to work: a qualitative study. BMJ Open 2012;**2**(5).
12. Cohen D, Winstanley SJ, Greene G. Understanding doctors' attitudes towards self-disclosure of mental ill health. Occupational Medicine 2016;**66**(5):383-89.
13. Department of Health. Mental health and ill health in doctors London: Department of Health, 2008.
14. Gold KJ, Andrew LB, Goldman EB, et al. "I would never want to have a mental health diagnosis on my record": A survey of female physicians on mental health diagnosis, treatment, and reporting. . General hospital psychiatry 2016;**43**:51-57.
15. Baird B, Charles A, Honeyman M, et al. Understanding pressures in general practice. 2016.
16. Hobbs FR, Bankhead C, Mukhtar T, et al. Clinical workload in UK primary care: a retrospective analysis of 100 million consultations in England, 2007–14. The Lancet 2016.
17. Doran N, Fox F, Rodham K, et al. Lost to the NHS: a mixed methods study of why GPs leave practice early in England. Br J Gen Pract 2016;**bjgpfeb**.
18. Firth-Cozens J. A perspective on stress and depression. . In: J. Cox JK, A. Hutchinson & P. McAvoy, ed. Understanding doctors' performance. Oxford: Radcliffe Publishing., 2006:22–25.

For peer review only - http://bmjopen.bmj.com/site/about/guidelines.xhtml

19. Firth-Cozens J. *A Little too Much, in How to Survive in Medicine: Personally and professionally*. Oxford: Wiley-Blackwell, 2010.
20. Doran N, Fox F, Rodham K, et al. Lost to the NHS: a mixed methods study of why GPs leave practice early in England. British journal of general practice 2016;**bjgpfeb-2016**.
21. Hippisley-Cox J, Vinogradova Y. Trends in Consultation Rates in General Practice 1995 to 2008: Analysis of the QResearch database. . London: NHS Information Centre:, 2009.
22. King's Fund. Improving the quality in General Practice. . London: King's Fund, 2011.
23. Ahola K, Hakanen J. Job strain, burnout, and depressive symptoms: A prospective study among dentists. J Affect Disorders 2007;**104**:103 - 10.
24. Clarke J, O'Sullivan Y, Maguire N. A study of self-care among Irish doctors. Irish medical journal 1997;**91**(5):175-76.
25. DoH. National Survey of General Practitioner Job Satisfaction. 2012.
26. Dyrbye LN, Varkey P, Boone SL, et al. Physician satisfaction and burnout at different career stages. Mayo Clin Proc 2013;**88**(12):1358-67.
27. Firth-Cozens J. Predicting stress in general practitioners: 10 year follow up postal survey. British Medical Journal of Addiction Medicine 1997;**315**:34–35.
28. Fram SM. The constant comparative analysis method outside of grounded theory. . The Qualitative Report 2013;**18**(1):1.
29. Sandewaloski MS. Sample size in qualitative research. Res Nurs Health 1995;**18**:179-83.
30. Thompson N, Corbett, SS, Welfare M. A qualitative study of how doctors use impression management when they talk about stress in the UK. International Journal of Medical Education 2013;**4**:236-46.
31. Wiegner L, Hange D, Björkelund C, Ahlborg GJ. Prevalence of perceived stress and associations to symptoms of exhaustion, depression and anxiety in a working age population seeking primary care-an observational study. BMC Fam Pract 2015;**16**(38).
32. NHS England. NHS GP Health Service. Secondary NHS GP Health Service 2017. https://www.england.nhs.uk/gp/gpfv/workforce/health-service/.
33. Van Roy K, Vanheule S, Inslegers R. Research on Balint groups: a literature review. Patient Educ Couns 2015;**98**(6):685–94
34. Benson J, Magraith K. Compassion fatigue and burnout: the role of Balint groups. Australian family physician 2005;**34**(6):497-8.
35. Gishen F, Gill D. Introducing Schwartz rounds at UCL Medical School. Secondary Introducing Schwartz rounds at UCL Medical School 2014. https://uclmsnews.wordpress.com/2014/09/29/introducing-schwartz-rounds-at-ucl-medical-school/.
36. BMA. Are you concerned about a colleague? Secondary Are you concerned about a colleague? 2017. https://www.bma.org.uk/advice/work-life-support/your-wellbeing/concerned-about-a-colleague.

For peer review only - http://bmjopen.bmj.com/site/about/guidelines.xhtml

Table 1: Participant demographics

GP Characteristics	N=47
	N
Sex (female)	33
Age	
20-29	1
30-39	12
40-49	20
50-59	14
Group	
1	19
2	9
3	11
4	8
Years since qualified	
<10	19
No of sessions contracted per week	
<5 sessions (mean actual hours worked)	12 (15)
>5 sessions (mean actual hours worked)	32 (38)
Fully retired	3
Mean size of practice	12624
Range	3,600 – 37,000
Employment status	
Partner	17
Salaried	11
Locum	5
Registrar	4
Retired	3
Sick leave	5
More than one role	2

For peer review only - http://bmjopen.bmj.com/site/about/guidelines.xhtml

Box 1. Interview topic guide

Introduction and background

- Describe average working day in practice (hours, surgery, home-visits) and any additional responsibilities

Current well-being

- Describe average working day in practice (hours, surgery, home-visits) and any additional responsibilities
- Explore current well-being, feelings about work, levels of stress, work-life balance
- Explore causes of stress/distress (work load, hours, admin, clinical caseloads, organisational issues, lack of support, personal issues, pre-existing mental health symptoms)
- Explore reasons for early retirement/sickness (if relevant)

Managing stress

- Explore how GPs manage their workload/stress in their day-to-day work life, what they do to relax, to look after themselves (self-care strategies: supportive relationships, sport, exercise relaxation techniques)
- Explore relationship with colleagues and whether/how/if concerns are raised, how they are responded
- Explore if receive informal/formal supervision or mentor (1:1 or group) and experience/value of group
- Explore thoughts/feelings about seeking help, barriers to seeking help (stigma/shame, fears about confidentiality, uncertainty of where to go)

only

For peer review only - http://bmjopen.bmj.com/site/about/guidelines.xhtml

Consolidated criteria for reporting qualitative studies (COREQ): 32-item checklist

Developed from:
Tong A, Sainsbury P, Craig J. Consolidated criteria for reporting qualitative research (COREQ): a 32-item checklist for interviews and focus groups. *International Journal for Quality in Health Care*. 2007. Volume 19, Number 6: pp. 349 – 357

No. Item	Guide questions/description	Reported on Page #
**Domain 1: Research team and reflexivity**		
Personal Characteristics		
1. Interviewer/facilitator	Which author/s conducted the interview or focus group?	Page 5
2. Credentials	What were the researcher's credentials? E.g. PhD, MD	Page 6
3. Occupation	What was their occupation at the time of the study?	Page 1/6
4. Gender	Was the researcher male or female?	Page 1
5. Experience and training	What experience or training did the researcher have?	Page 6
Relationship with participants		
6. Relationship established	Was a relationship established prior to study commencement?	Page 5 .
7. Participant knowledge of the interviewer	What did the participants know about the researcher? e.g. personal goals, reasons for doing the research	Page 5
8. Interviewer characteristics	What characteristics were reported about the inter viewer/facilitator? e.g. Bias, assumptions, reasons and interests in the research topic	Page 6

For peer review only

**Domain 2: study design**		
Theoretical framework		
9. Methodological orientation and Theory	What methodological orientation was stated to underpin the study? e.g. grounded theory, discourse analysis, ethnography, phenomenology, content analysis	Page 6
Participant selection		
10. Sampling	How were participants selected? e.g. purposive, convenience, consecutive, snowball	Page 5
11. Method of approach	How were participants approached? e.g. face-to-face, telephone, mail, email	Page 5
12. Sample size	How many participants were in the study?	Page 5/6
13. Non-participation	How many people refused to participate or dropped out? Reasons?	None
Setting		
14. Setting of data collection	Where was the data collected? e.g. home, clinic, workplace	Page 5 .
15. Presence of non-participants	Was anyone else present besides the participants and researchers?	No
16. Description of sample	What are the important characteristics of the sample? e.g. demographic data, date	Page 5 &15
Data collection		
17. Interview guide	Were questions, prompts, guides provided by the authors? Was it pilot tested?	Page 16
18. Repeat interviews	Were repeat interviews carried out? If yes, how many?	No, due to lack of resources
19. Audio/visual recording	Did the research use audio or visual recording to collect the data?	Page 6

For peer review only

20. Field notes	Were field notes made during and/or after the interview or focus group?	No
21. Duration	What was the duration of the inter views or focus group?	Page 5
22. Data saturation	Was data saturation discussed?	Yes, page 6
23. Transcripts returned	Were transcripts returned to participants for comment and/or correction?	No, due to lack of resources
**Domain 3: analysis and findings**		
Data analysis		
24. Number of data coders	How many data coders coded the data?	Page 6
25. Description of the coding tree	Did authors provide a description of the coding tree?	No
26. Derivation of themes	Were themes identified in advance or derived from the data?	Page 6
27. Software	What software, if applicable, was used to manage the data?	Page 6
28. Participant checking	Did participants provide feedback on the findings?	No, due to lack of resources
Reporting		
29. Quotations presented	Were participant quotations presented to illustrate the themes/findings? Was each quotation identified? e.g. participant number	Pages 7-10
30. Data and findings consistent	Was there consistency between the data presented and the findings?	Yes
31. Clarity of major themes	Were major themes clearly presented in the findings?	Yes
32. Clarity of minor themes	Is there a description of diverse cases or discussion of minor themes?	Yes

For peer review only

BMJ Open

'Treading water but drowning slowly': What are GPs' experiences of living and working with mental illness and distress in England? A qualitative study

Journal:	*BMJ Open*
Manuscript ID	bmjopen-2017-018620.R1
Article Type:	Research
Date Submitted by the Author:	12-Oct-2017

Complete List of Authors:	Riley, Ruth; University of Birmingham, Institute of Applied Health Research; University of Bristol, Social and Community Medicine<br>Spiers, Johanna ; University of Bristol School of Social and Community Medicine<br>Chew-Graham, Carolyn; University of Keele, Primary Care and Health Sciences<br>Taylor, Anna ; University of Bristol, Social and Community Medicine<br>Thornton, Gail; University of Bristol, Social and Community Medicine<br>Buszewicz, Marta; University College London, Research Department of Primary Care and Population Health

**Primary Subject Heading**:	General practice / Family practice
Secondary Subject Heading:	Mental health, Qualitative research, Occupational and environmental medicine
Keywords:	General Practitioners, Depression, Anxiety, Distress, Chronic Stress, Suicidal Ideation

SCHOLARONE™
Manuscripts

‘Treading water but drowning slowly’: What are GPs’ experiences of living and working with mental illness and distress in England? A qualitative study

Ruth Riley

Lecturer

Institute of Applied Health Research

University of Birmingham

Birmingham

United Kingdom

r.riley@bham.ac.uk

0121 414 8549

Johanna Spiers

Senior Research Associate

Centre for Academic Primary Care

University of Bristol

United Kingdom

0117 33 14532

Js16447@bristol.ac.uk

Carolyn A. Chew-Graham

Professor of General Practice Research

Research Institute, Primary Care and Health Sciences

Keele University

Keele

United Kingdom

01782 734 717

c.a.chew-graham@keele.ac.uk

For peer review only

For peer review only - http://bmjopen.bmj.com/site/about/guidelines.xhtml

Anna Kathryn Taylor

Medical Student

Faculty of Health Sciences

University of Bristol

Bristol

United Kingdom

0117 331 6825

at0012@my.bristol.ac.uk

Gail A. Thornton

Patient and Public Contributor

Centre for Academic Primary Care

University of Bristol

United Kingdom

0117 9104917

gail@thorntonuk.com

Marta Buszewicz

Reader in Primary Care

Research Department of Primary Care and Population Health

University College London

London

United Kingdom

020 7794 0500 extn. 31016

m.buszewicz@ucl.ac.uk

Word count: 3,596

Abstract

Objectives. This paper provides an in-depth account of General Practitioners' (GPs) experiences of living and working with mental illness and distress, as part of a wider study

For peer review only

For peer review only - http://bmjopen.bmj.com/site/about/guidelines.xhtml

reporting the barriers and facilitators to help-seeking for mental illness and burnout, and sources of stress/distress for GP participants.

Design. Qualitative study using in-depth interviews with 47 GP participants. The interviews were audio-recorded, transcribed, anonymised and imported into NVivo11 to facilitate data management. Data were analysed using a thematic analysis employing the constant comparative method.

Setting. England

Participants. A purposive sample of GP participants who self-identified as: 1) currently living with mental distress, 2) returning to work following treatment, 3) off sick or retired early as a result of mental distress or 4) without experience of mental distress. Interviews were conducted face to face or over the telephone.

Results. The findings report GP participants' in-depth experiences of distress and mental illness with many recollecting their distressing experiences and significant psychological and physical symptoms relating to chronic stress, anxiety, depression and/or burnout, and almost half articulating thoughts of suicide. Many talked about their shame, humiliation and embarrassment at their perceived inability to cope with the stresses of their job and/or their symptoms of mental illness.

Conclusions. These findings paint a concerning picture of the situation affecting primary care doctors, with participants' accounts suggesting there is a considerable degree of mental ill health and reduced wellbeing amongst GPs. The solutions are complex and lie in prevention and provision. There needs to be greater recognition of the components and cumulative effect of occupational stressors for doctors, such as the increasing workload and the clinical and emotional demands of the job, as well as the need for a culture shift within medicine to more supportive and compassionate work environments.

Keywords: Qualitative, General Practitioners, Depression, Anxiety, Distress

Strengths and limitations of this study

- Few studies employing qualitative methods, using in-depth interviews, have been utilised to examine this topic and depict the experience of distress amongst GPs. This study carried out 47 interviews with GPs from across England and contributes to a growing body of research illuminating and examining the experience of living with chronic stress and distress amongst this population of doctors

- Participants were self-selecting, which may be perceived as a limitation; however, the recruitment of participants ensured that the sample was varied in terms of age, gender, number of years as practising GPs, level of seniority/employment status in

For peer review only - http://bmjopen.bmj.com/site/about/guidelines.xhtml

the practice, and geographical location. This variation provided a range of perspectives; however, uniform differences between the groups were not observed.

- The researchers did not employ respondent validation; however, the second coders included academic GPs and team members with lived experience of mental illness which afforded some checks and balances to the validity of the analytic process, interpretation of data and generalizability of the research findings.

- In recruiting individuals with experience of mental illness and burnout, we also included the perspectives of GPs who had no disclosed history of mental illness, which enables the data to be more widely transferable to the UK GP population

- The multi-disciplinary research team independently analysed a sub-set of transcripts in order to contribute to the generation and refinement of codes to maximise rigour. Emergent themes were subsequently discussed by the whole team to ensure credibility and confirmability.

INTRODUCTION

Compared with the general population, doctors, including general practitioners (GPs) working in primary care, experience high levels of psychological distress linked to chronic stress, anxiety, depression and burnout [1-4]. These doctors also have higher rates of mental illness and suicide [5-8]. Evidence from industrialized countries (e.g. UK, USA, Australia), indicates that suicide rates are higher amongst GPs, psychiatrists and anaesthetists compared with other medical specialities [6 8]. Doctors undergoing fitness for practice investigations are at particularly high risk of suicide [9]. Compared with the general population, doctors also have higher rates of drug and alcohol misuse and dependency, with a higher rate of misuse of prescription medicines [10]. Psychological difficulties or distress experienced by doctors can be further compounded by self and societal stigmatising associated with mental illness, with evidence that doctors avoid or delay disclosure and help-seeking due to concerns about loss of confidentiality, denial of illness and logistical barriers to accessing support [11-15]. The prevailing hegemonic culture amongst medical practitioners and non-disclosure of vulnerability, with shame attached to mental health problems or psychological difficulties and the consequent perceived inability to cope within the job, also hinders help-seeking [4 11 16 17]. It has been suggested that personality characteristics such as perfectionism and obsessionality, whilst being positively associated with hard-working and conscientious professionals, may play a role in predisposing doctors towards chronic stress and/or mental ill-health [4].

The increasing prevalence of mental ill-health amongst the medical profession, including GPs, has been attributed to a number of factors, most notably the escalating occupational stresses related to workload, long hours and the emotional demands of the job [4 18-23]. In

For peer review only - http://bmjopen.bmj.com/site/about/guidelines.xhtml

addition, sharp increases in patient demand [19 24] and transfer of care from specialist to primary care [25], as well as reductions in resources [25], have resulted in the pressure on primary care in England being at its highest ever. This pressure is predicted to increase in the future [25]. Findings such as these highlight the potentially stressful context in which GPs are currently working, leaving many vulnerable to distress and/or mental health challenges [26 13 14 23].

Evidence of the extent of mental ill-health and burnout amongst GPs is largely derived from survey data, with some small-scale qualitative studies exploring the topic [13 27-31]. To date, few studies have explored the experiences and accounts of GPs living and working with enduring levels of stress, mental ill-health and psychological distress using in-depth qualitative methods. The employment of in-depth interviews in this study allowed GP participants to describe the full extent and complexity of their distress. In addition, by providing the 'patient's view' [32], qualitative findings can fully elucidate details and context about phenomena in a way that quantitative work cannot [33]. This paper reports GPs' detailed experiences of living and working with mental illness and emotional distress, as part of a wider study reporting the sources of stress and distress[23] for GPs and the barriers and facilitators to help-seeking for mental illness and burnout [14] amongst this medical population.

METHODS

This multi-centre, qualitative study employed in-depth interviews with 47 GPs in England. Information about the study was advertised through professional publications such as Pulse, study newsletters, social media and national and local GP networks (including Local Medical Committees or LMCs) in Bristol, Manchester and London. A sub-sample (n=12) was recruited through a specialist treatment service. GPs interested in taking part were asked to self-select into the following groups: 1: Living with anxiety, depression, stress and/or, burnout; 2: Returning to work following treatment; 3: Off sick or retired early due to psychological ill-health 4: No mental ill-health. GPs were offered reimbursement of £80 to recognise the time for their participation.

GPs who expressed an interest in participating were purposively sampled to represent as even a spread as possible across these four groups, although the largest number of participants were in Group 1. We intended to recruit approximately 10 participants per group. However, the majority of GPs who contacted us self-selected into group one and, due to the emergent rich data, continued recruitment to this group and further exploration of emerging themes was justified in meeting the study's aims and objectives. We endeavored to recruit more participants into groups two and four using targeted publicity information, but because of time constraints those groups remained marginally under-recruited. In the event, many GPs who identified as living with no stress reported as having had experiences of stress and distress at some juncture in their career. More female GPs contacted the study team expressing an interest in participating, and therefore the disparity in numbers interviewed reflects this. The iterative process of recruitment, sampling and analysis ensured that emerging concepts and themes could be tested out amongst participants with different characteristics (e.g. partners vs locum GPs).

For peer review only - http://bmjopen.bmj.com/site/about/guidelines.xhtml

Face-to-face or telephone interviews lasting between 27 and 126 minutes (mean = 69 minutes) were conducted between April and September 2016. The majority of interviews were conducted in participants' homes. The in-depth interviews were conducted by two authors (JS, RR) using a flexible topic guide (see Box 1). This was informed by the existing literature, input from GPs on the study team and PPI consultation exercises conducted with GP networks prior to obtaining funding. The interviews were audio-recorded, transcribed, anonymised and imported into NVivo11 to facilitate data management. Analysis and data collection were conducted iteratively. A thematic analysis was performed involving a process of constant comparison between cases [34]. Analysis commenced with JS generating an initial coding framework grounded in the data, which was added to and refined, with material regrouped and recoded as new data were gathered. Codes were gradually built into broader categories through comparison across transcripts, and higher-level recurring themes were developed until data saturation was reached when no new themes were arising from the data[35]. Reflexivity was employed throughout the research. Both interviewers were experienced qualitative researchers who both reflected on and discussed the impact of the data on their cognitive and emotional sensing throughout the study. Both researchers also discussed and made explicit how their epistemological (JS with a background in psychology and RR in medical sociology) and experiential backgrounds may have oriented the data collection and analytic process.

Members of the multi-disciplinary research team independently analysed a sub-set of transcripts in order to contribute to the generation and refinement of codes to maximise rigour. Emergent themes were discussed by the whole team to ensure credibility and confirmability.

RESULTS

Willing participants were purposively sampled from GPs who expressed an interest in the study, based on demographic and practice characteristics as well as self-selected group type. The characteristics of the participants are found in Table 1.

<<<<insert table 1 here>>>>>

For this paper we will present the following five main themes and corresponding sub themes relating to:

1. Burnout - loss of empathy or compassion fatigue, feeling empty and/or exhausted, difficulty making decisions, low job satisfaction and loss of enjoyment in seeing patients.
2. Anxiety - psychological symptoms relating to anxiety, including feeling anxious or on edge, worrying, irritability, inability to relax and physical symptoms related to anxiety such as panic attacks, hyperventilating, palpitations, sweating and nausea.
3. Depression – distressed at work, insomnia and exhaustion, negative thoughts, low self-esteem, irritability and anger.

For peer review only - http://bmjopen.bmj.com/site/about/guidelines.xhtml

4. Suicidal ideation - thoughts of suicide.
5. Shame, as well as feelings of failure and humiliation, resulting from a perceived lack of resilience and inability to cope.

It should be noted that some participants described or disclosed symptoms that could be clearly attributed to chronic stress, anxiety, depression and burnout. Other participants described or framed their symptoms in terms of diagnostic concepts associated with these categories, whilst others talked about their feelings and emotional state without necessarily labelling them. Notably, the symptomology was identified across all groups including Group 4, that is individuals who self-identified as having no mental health concerns. The themes also applied to participants irrespective of seniority or role in the practice, age or gender. There was no clear distinction in terms of presentation or severity of symptoms between the groups and so no typology could be developed along these lines.

1. Symptoms of Burnout

Some participants reported feeling a loss of empathy or compassion fatigue, feeling empty, exhaustion, difficulty making decisions, low job satisfaction and loss of enjoyment for their work, using language which highlights the depth of the feelings:

I'd lost my empathy with my patients. You know, and er I'm just – I was just – you're just like a ketchup bottle in a production line […] But from a personal point of view I'm still feeling a bit washed out by it, you know, I feel a little bit empty. You know, I feel like I'm burnt out really a bit, to be honest. (P25, Male Salaried)

I was saying all the right things but […] I felt quite detached from it. (P27, Female Salaried)

The following participant describes how feeling overworked can lead to a reduced ability to offer compassion and support to patients:

If you find yourself working too many sessions, I can, you know, I feel it, and you almost feel like you lose the milk of human kindness. And you can't do that job if you're not (pause) at your best. (P21, Female, Salaried and Locum)

2. Symptoms of Anxiety

The majority of participants, including those who self-identified as having no mental health concerns (Group 4), described psychological symptoms relating to anxiety, including feeling on edge or hyper-alert, persistent worrying, irritability and an inability to relax. The following examples demonstrate how difficult and distressing these symptoms and feelings were for some participants to negotiate:

For peer review only - http://bmjopen.bmj.com/site/about/guidelines.xhtml

Everything was running around in my mind at 200 miles an hour. I was in sort of hyper drive and, yeah, you wake up to super stimulated, super awake, super, you know, on edge and anxious. (P36, Male Partner)

Many participants also described physical symptoms related to their anxiety, such as panic attacks, hyperventilating, palpitations, sweating, and nausea:

Erm (pause) and I, and I was getting quite bad chest pain, palpitations, I was feeling anxious in many circumstances, like the school run, just meeting people, going to meetings and it was becoming incapacitating where it felt like somebody had got a knife in my chest all the time. And I knew it wasn't anything to do with my heart. (P31, Female Locum)

3. Symptoms of Depression

Many GP participants described symptoms relating to depression, which included: feeling distressed at work, low mood, hopelessness, sleep problems, low self-esteem, guilt, restlessness, fatigue and suicidal feelings. Some participants employed a range of poignant and powerful metaphors which illustrate the extent of those difficult emotions:

And, to be honest with you, that kind of er – the best way that I could describe that is that it's kind of like a dark shadow that's in the corner of the room […] I basically felt like I was treading water but drowning slowly. (P45, Male Salaried)

Distressed at work:

Many participants recalled their experiences of feeling distressed, anxious and tearful, with some describing breaking down at home, after or during work or in front of patients:

I mean there were times certainly when I arrived at work and just sat outside and cried before I even went in the building. And I often cried on the way home. I'd often cry when I came in from work, um just collapse in a heap, and get to bed actually crying. (P31, Female Locum)

I remember once actually sitting under the desk, hugging my knees to my chest, I was so anxious. (P25, Male Salaried)

I would just end up in tears all the time at the end of each session. (P53, Female Salaried)

Insomnia and exhaustion:

Amongst the myriad of symptoms, insomnia and exhaustion were a commonplace experience for study participants:

Um - so I wasn't sleeping and I wasn't - wasn't eating very well, and - and it just all sort of took its toll. (P23, Female Salaried)

For peer review only - http://bmjopen.bmj.com/site/about/guidelines.xhtml

It's very hard to function when you're not ever getting refreshing sleep […] And I just wouldn't stop. And absolute, incapacitating terror. Um - So, but one's having um — night, night terrors maybe two, three, four times a week. Sometimes more than one a night. (P24, Female Partner)

Many GP participants detailed their exhaustion, which was often linked to insomnia and could be symptomatic of their anxiety, depression and/or feeling burnt out, demonstrating the ways in which different elements of GP experience combine to add to distress:

Probably like sort of swimming through treacle, so everything seemed - um - much harder. (P38, Male Partner)

So like you can't, you can't avoid that fatigue. You might be able to put it off a bit (laughs) but you're going to pay interest on it. (P7, Female Locum)

Negative thoughts/low self-esteem:

Some participants talked about grappling with cycles of negative thoughts, symptomatic of low self-esteem and a common symptom of depression, as this participant illustrates:

And so then I'm really struggling, because I'm struggling with my internal dialogue saying, you know, "Not good enough, could be doing a better job. Not really, not really er making people better." […] persistent negative cycles of er (pause), yeah, of negative thoughts about yourself (P20, Male Partner)

Irritability and anger:

Some study participants described feeling irritable and sometimes angry; a possible symptom of depression in this context:

But I think, towards the end when I stopped locum-ing again, I was beginning to feel very tired as a result and that it was taking more out of me than it ought to really, and feeling um, yeah, feeling more irritable. (P47, Female on sick leave)

This account demonstrates how feeling stressed and irritable can sometimes manifest itself within a work context:

…it was fairly um traumatic really, I have to say…. I think I'd become quite stressed at work actually and I'd lost my temper in meetings at times and um nobody quite knew what to do about it. (P39, Male Partner)

4. **Suicidal ideation**

For peer review only - http://bmjopen.bmj.com/site/about/guidelines.xhtml

Almost half of the GP participants described having had suicidal thoughts, with the following participants reflecting on occasions when they had contemplated and ruminated on ways of taking their own lives:

And it just came on me like a wave. And then I started thinking, "I'm going to jump onto those train tracks." (P45, Male Salaried)

And um constant ruminations on suicide and doing myself in. And the trick with suicide is to persuade yourself that those around you would be better off without you, um and I got to that point and I decided on ways that I might (pause) do myself in and ruminated about that all the time. Just, it just filled my waking and sleeping nights. (P20, Male Partner)

This latter data extract demonstrates how suicidal thoughts can be both constant and normalised for some GPs.

The following GP reflects on the advice she gives to her patients who express suicidal thoughts and how her feelings mirrored her patients' expressed hopelessness and despair, again showing the ways in which GP experiences can combine to add to distress:

Um, and then I think the hard thing, the worst thing I think I found was when people were feeling suicidal, and they were saying they were feeling suicidal and why they were feeling suicidal. And normally I'd be like, "Look, you know, it's temporary, it'll get better. These are the things that we can do," but I just sat there thinking, "Well, you're probably right, it is rubbish," you know. (P27, Female Partner)

5. Shame and feelings of failure

Perhaps symptomatic of the stigma attached to mental illness and the culture of invulnerability within medicine, some GP participants described how they felt ashamed, embarrassed, humiliated and a sense of having failed, due to their perceived lack of resilience and inability to cope. These quotations illustrate the depth of that emotion for two of the participants:

And you do feel a huge sense of shame, huge sense of shame. Because even though I treat patients day in, day out with anxiety and depression, it felt like this was my not coping. And I was thinking, "Why are the partners all able to cope with this but not me?" and that sort of thing. So it was all rolled into one really. (P4, Female Partner)

Er (sigh) yeah, I think for me it was very embarrassing. Didn't want to admit it. Couldn't believe that like I'd got to this stage. Cos I'd never been someone like that […] yeah, I did find it really hard, I think, to actually accept that this had happened. Um (pause) yeah, bit of a failure? I suppose, in a way. Kind of feel like you're not strong enough? (P44, Female Registrar)

The participant below highlights how the denial and intolerance of mental ill health amongst some colleagues can enter into an individual's internal dialogue, thus compounding existing feelings of shame:

For peer review only - http://bmjopen.bmj.com/site/about/guidelines.xhtml

'Yeah, well you don't get ill, do you? You know, you, you refuse to accept any illness in yourself, don't you? It's just a question of that's why doctors present late with illnesses because they don't like to make a fuss. We see it as a sign of weakness, which I think probably is why we are intolerant of illness in our partners, because we're doctors. And I think it's just part of the sort of macho thing which is first drummed into you at medical school and the end product of that generation, which – if you can't take the heat get out of the kitchen, type thing'. (P49, Female, Retired)

DISCUSSION

The accounts of the 47 participants in this study suggest there may be an undetermined population of GPs who are living and working with poor mental health. Participants vividly described and recollected their distressing experiences and troubling symptoms, with almost half of the sample articulating thoughts of suicide. The GP participants described numerous troubling psychological and physical symptoms relating to chronic stress, anxiety, depression and/or burnout with several breaking down at work – crying, feeling despair and many contemplating suicide. Many also talked about their shame, humiliation and embarrassment at their perceived inability to cope with the stresses of their job and/or their symptoms of mental ill-health. The experiences and feelings of many GP participants depict a dispiriting emotional landscape, with notable signs of poor mental health and striking levels of distress.

There has been a paucity of in-depth qualitative research exploring this topic area with doctors, including GPs [14]. This study highlights that many GPs are living *and working* with extreme levels of emotional distress. A notable proportion of participants vividly recalled and articulated both fleeting and recurring thoughts of suicide, which is reflective of the extent of individuals' distress and suffering. These candid, evocative and sometimes disturbing accounts, some hitherto unvoiced, reveal a population of doctors with generally very poor mental health.

The findings from this study and the related papers[14 23] were based on in-depth interviews with 47 GPs from across England and contribute to a growing corpus of research illuminating and examining the causes, experiences and impact of chronic stress and distress amongst GPs [20 36]. The participants were self-selecting, which may be seen as a limitation; however, the recruitment of participants ensured that the sample was varied in terms of age, gender, number of years as practising GPs, level of seniority/employment status in the practice, and geographical location. In addition to recruiting individuals with self-reported mental ill-health and burnout, we also included the perspectives of GPs who had no disclosed history of mental health problems, which enables the data to be more transferable to the wider GP population. However, despite this heterogeneity within the sample, we did not observe any marked differences between the groups, suggesting a certain uniformity of distress for the group despite demographic differences.

For peer review only - http://bmjopen.bmj.com/site/about/guidelines.xhtml

These findings present a concerning picture of psychological difficulties and reduced wellbeing affecting primary care doctors. The experience of chronic stress and anxiety in GPs (e.g. feeling tense, restless, anxious, worried or having disrupted sleep, panic attacks, hyperventilating, palpitations, sweating, and nausea) appears commonplace, including in those who did not identify themselves as having mental ill-health. Our findings reflect the evidence that doctors frequently normalise, minimise, deflect or downplay how stressed they feel, which may preclude help-seeking and the availability of appropriate support [37]. Strikingly, one GP participant normalised the way they routinely ruminated on their suicidal thoughts, with other participants indicating that they were living and coping on a day-to-day basis with symptoms of chronic stress, anxiety and depression, ranging from sleeplessness to persistent anxiety. Evidence indicates that chronic stress negatively impacts on physical health and is a key risk factor for mental illness, including anxiety and depression, and can lead to the adoption of maladaptive coping mechanisms such as nicotine, alcohol, and illicit drug use [38].

Normalising and acknowledging chronic stress as an occupational hazard associated with being a doctor is helpful, but only if solutions are sought which focus on prevention and the provision of support to maintain the health and wellbeing of frontline clinicians. Since January 2017, GPs and GP trainees in England suffering from mental ill health and addiction have been able to access free support through the National Health Service's GP Health Service [39]. Further research and evaluation must be undertaken to assess the effectiveness of this service. The emphasis now needs to focus on preventing mental ill health and improving the support available to doctors. Access to personal, relational and environmental resources are protective factors for good mental health and wellbeing and impact on individuals' resilience [40]. Crucially, collegial support is a protective factor for good mental health - support from mentors, supervisors and colleagues is associated with resilience and reduced sickness [22]. Balint groups or similarly structured group work or supervision continue to be employed in general practice and are valued by GPs [41], yet are not used across all practices. Individual or group supervision aims to provide a safe and supportive space where staff can openly discuss the pressures and emotional challenges of their work and may, as previous evidence suggests, provide GPs with the support they need whilst offering protection against compassion fatigue and burnout [42]. Its wider use may need to be considered.

Tackling the culture of invulnerability early on in medical training is also key. Schwartz Centre Rounds®, for instance, are currently being piloted in medical schools with early evidence supporting their value [43]. First5®[44], buddy systems, and access to regular supervision or mentorship can also offer support and a reflective space. The British Medical Association (BMA) have produced guidance on recognising signs of mental distress amongst colleagues with suggestions about what action to take [45]. Perhaps caution needs to be applied to the over-reliance on personal resilience as a solution as this may shift the responsibility for maintaining good mental health entirely on to the individual. While self-care is paramount in maintaining good mental and physical health, it is not solely the responsibility of individual doctors to look after their mental health; employers and

For peer review only - http://bmjopen.bmj.com/site/about/guidelines.xhtml

colleagues need to engender a more supportive and compassionate culture. Doctors are not invulnerable to suffering, most especially their own.

In the light of the findings of this study, we recommend the following:

- Greater recognition of the components and cumulative effect of the occupational stressors for doctors, such as increasing workload and the clinical and emotional demands of the job.
- A culture change within medicine, with a shift to more supportive and compassionate work cultures.
- The de-stigmatising of mental ill-health and the recognition of GPs' experiences and emotional responses as symptomatic of occupational stress as opposed to any perceived failure to cope in the individual.

Conclusion

These findings, together with those from the wider study[14 23], paint a concerning picture of the situation affecting primary care doctors, with participants' accounts suggesting there is a population of GPs experiencing a considerable degree of mental ill health, and overall reduced wellbeing amongst GPs. The solutions are complex and prevention lies in addressing the systemic causes of stress (related to diminishing resources, increased workload, etc.). In addition to recognising the components and cumulative effect of occupational stressors for doctors, such as the increasing workload and the clinical and emotional demands of the job, there is need for a culture shift within medicine to more supportive and compassionate work environments. Medical schools, medical institutions and individuals need to address and tackle the stigma of mental illness and engender a greater tolerance and acceptance of invulnerability.

Author Contribution:

RR, JS, CCG, MB– substantial contributions to conception and design, acquisition of data, or analysis and interpretation of data; drafting the article or revising it critically for important intellectual content; and final approval of the version to be published.

AT & GT - analysis and interpretation of data; drafting the article or revising it critically for important intellectual content; and final approval of the version to be published.

Acknowledgements: The authors would like to thank all volunteer participants who kindly gave their time to share their experiences of working in English general practice; and collaborators Dr Clare Gerada and Dr Chris Manning for endorsing this study, providing valuable comments on the funding application and assisting with recruitment.

Competing interests: None

Funding: The study was funded by NIHR School for Primary Care Research

For peer review only - http://bmjopen.bmj.com/site/about/guidelines.xhtml

Department of Health Disclaimer: The views and opinions expressed therein are those of the authors and do not necessarily reflect those of the NIHR School for Primary Care Research, NIHR, NHS or the Department of Health.

Ethical approval: Ethical approval was granted by the South West - Frenchay Research Ethics Committee (reference number: 15/SW/0350)

Data sharing statement: This study has not received ethical approval to share confidential data with any third party other than the study research team.

REFERENCES

1. Chopra SS, Sotile WM, Sotile MO. Physician burnout. JAMA 2004;**291**(5):633-33.
2. Merlo LJ, Singhakant, S., Cummings S.M., and Cottler L.B. . Reasons for Misuse of Prescription Medication Among Physicians Undergoing Monitoring by a Physician Health Program. Journal of addiction medicine 2013;**7**(5):349-53.
3. Orton P, Orton C, Pereira Gray D. Depersonalised doctors: a cross-sectional study of 564 doctors, 760 consultations and 1876 patient reports in UK general practice. BMJ Open 2012;**2**(1).
4. Brooks SK, Gerada, C., Chalder, M. Review of literature on the mental health of doctors: Are specialist services needed? Journal of Mental Health 2011:1-11.
5. Dobbin A. Burnt out or fired up? Helping recovery in distressed patients can increase our own resilience. Br J Gen Pract 2014;**64**(627):497-98.
6. Hawton K, Clements A, Sakarovitch C, et al. Suicide in doctors: a study of risk according to gender, seniority and specialty in medical practitioners in England and Wales, 1979–1995. Journal of Epidemiology and Community Health 2001;**55**(5):296-300.
7. Richings JC, Khara, G. S. & Mcdowell, M. Suicide in young doctors. . British Journal of Psychiatry 1986;**149**:475-78.
8. Schernhammer ES, Colditz GA. Suicide Rates Among Physicians: A Quantitative and Gender Assessment (Meta-Analysis). American Journal of Psychiatry 2004;**161**(12):2295-302.
9. GMC. Doctors who commit suicide while under GMC fitness to practise investigation. 2014.
10. Brooks SK, Chalder T, Gerada C. Doctors vulnerable to psychological distress and addictions: treatment from the Practitioner Health Programme. . J Ment Health 2011;**20**:157–64.
11. Henderson M, Brooks SK, del Busso L, et al. Shame! Self-stigmatisation as an obstacle to sick doctors returning to work: a qualitative study. BMJ Open 2012;**2**(5).
12. Cohen D, Winstanley SJ, Greene G. Understanding doctors' attitudes towards self-disclosure of mental ill health. Occupational Medicine 2016;**66**(5):383-89.
13. Thompson WT, Cupples ME, Sibbett CH, et al. Challenge of culture, conscience, and contract to general practitioners' care of their own health: qualitative study. British Medical Journal 2001;**323**:728–31.
14. Spiers J, Buszewicz M, Chew-Graham CA, et al. Barriers, facilitators, and survival strategies for GPs seeking treatment for distress: a qualitative study. Br J Gen Pract 2017.
15. Cheshire A, Hughes J, Lewith G, et al. GPs' perceptions of resilience training: a qualitative study. Br J Gen Pract.
16. Department of Health. Mental health and ill health in doctors London: Department of Health, 2008.
17. Gold KJ, Andrew LB, Goldman EB, et al. "I would never want to have a mental health diagnosis on my record": A survey of female physicians on mental health diagnosis, treatment, and reporting. . General hospital psychiatry 2016;**43**:51-57.
18. Baird B, Charles A, Honeyman M, et al. Understanding pressures in general practice. 2016.

For peer review only - http://bmjopen.bmj.com/site/about/guidelines.xhtml

19. Hobbs FR, Bankhead C, Mukhtar T, et al. Clinical workload in UK primary care: a retrospective analysis of 100 million consultations in England, 2007–14. The Lancet 2016.
20. Doran N, Fox F, Rodham K, et al. Lost to the NHS: a mixed methods study of why GPs leave practice early in England. Br J Gen Pract 2016;**bjgpfeb**.
21. Firth-Cozens J. A perspective on stress and depression. . In: J. Cox JK, A. Hutchinson & P. McAvoy, ed. Understanding doctors' performance. Oxford: Radcliffe Publishing., 2006:22–25.
22. Firth-Cozens JH, J. *A Little too Much, in How to Survive in Medicine: Personally and professionally*. Oxford: Wiley-Blackwell, 2010.
23. Riley R, Spiers J, Buszewicz M, et al. 'What are the Sources of Stress and Distress for General Practitioners Working in England? A Qualitative Study' BMJ Open 2017.
24. Hippisley-Cox J, Vinogradova Y. Trends in Consultation Rates in General Practice 1995 to 2008: Analysis of the QResearch database. . London: NHS Information Centre:, 2009.
25. King's Fund. Improving the quality in General Practice. . London: King's Fund, 2011.
26. Brooks SK, Gerada C, Chalder M. Review of literature on the mental health of doctors: Are specialist services needed? Journal of Mental Health 2011:1-11.
27. Ahola K, Hakanen J. Job strain, burnout, and depressive symptoms: A prospective study among dentists. J Affect Disorders 2007;**104**:103 - 10.
28. Clarke J, O'Sullivan Y, Maguire N. A study of self-care among Irish doctors. Irish medical journal 1997;**91**(5):175-76.
29. DoH. National Survey of General Practitioner Job Satisfaction. 2012.
30. Dyrbye LN, Varkey P, Boone SL, et al. Physician satisfaction and burnout at different career stages. Mayo Clin Proc 2013;**88**(12):1358-67.
31. Firth-Cozens J. Predicting stress in general practitioners: 10 year follow up postal survey. British Medical. Journal of addiction medicine 1997;**315**:34–35.
32. Armstrong D. The patient's view. Social science & medicine 1984;**18**(9).
33. Smith JA. *Qualitative psychology: A practical guide to research methods.*Sage., 2007.
34. Fram SM. The constant comparative analysis method outside of grounded theory. The Qualitative Report 2013;**18**(1):1.
35. Sandewaloski MS. Sample size in qualitative research. Res Nurs Health 1995;**18**:179-83.
36. Fisher RFR, Croxson CHD, Ashdown HF, et al. GP views on strategies to cope with increasing workload: a qualitative interview study. British Journal of General Practice 2017.
37. Thompson N, Corbett, SS & Welfare, M. A qualitative study of how doctors use impression management when they talk about stress in the UK. International Journal of Medical Education 2013;**4**:236-46.
38. L W, Hange D, Björkelund C, et al. Prevalence of perceived stress and associations to symptoms of exhaustion, depression and anxiety in a working age population seeking primary care-an observational study. BMC Fam Pract 2015;**16**(38).
39. NHS England. NHS GP Health Service. Secondary NHS GP Health Service 2017. https://www.england.nhs.uk/gp/gpfv/workforce/health-service/.
40. Cusack L, Smith M, Hegney D, et al. Exploring environmental factors in nursing workplaces that promote psychological resilience: constructing a unified theoretical model? Front Psychol 2016;**7**:1-8.
41. Van Roy K, Vanheule S, R I. Research on Balint groups: a literature review. Patient Educ Couns 2015;**98**(6):685–94.
42. Benson J, Magraith K. Compassion fatigue and burnout: the role of Balint groups. Australian family physician 2005;**34**(6):497-8.
43. Gishen F, Gill, D. Introducing Schwartz rounds at UCL Medical School. Secondary Introducing Schwartz rounds at UCL Medical School 2014. https://uclmsnews.wordpress.com/2014/09/29/introducing-schwartz-rounds-at-ucl-medical-school/.

For peer review only - http://bmjopen.bmj.com/site/about/guidelines.xhtml

44. RCGP. http://www.rcgp.org.uk/membership/about-rcgp-membership/join-rcgp-newly-qualified-gps.aspx. 2017
45. BMA. Are you concerned about a colleague? Secondary Are you concerned about a colleague? 2017. https://www.bma.org.uk/advice/work-life-support/your-wellbeing/concerned-about-a-colleague.

Table 1: Participant and practice characteristics

**GP Characteristics**	**N=47**
	N
Sex (female)	33
Age	
20-29	1
30-39	12
40-49	20
50-59	14
Group	
1	19
2	9
3	11
4	8
Years since qualified	
<10	19
No of sessions contracted per week	
<5 sessions (mean actual hours worked)	12 (15)
>5 sessions (mean actual hours worked)	32 (38)
Fully retired	3
Mean size of practice	12624
Range	3,600 – 37,000
Employment status	
Partner	17
Salaried	11
Locum	5
Registrar	4
Retired	3
Sick leave	5
More than one role	2

For peer review only

Box 1. Interview topic guide

Introduction and background

- Describe average working day in practice (hours, surgery, home-visits) and any additional responsibilities

Current well-being

- Explore current well-being, feelings about work, levels of stress, work-life balance
- Explore causes of stress/distress (work load, hours, admin, clinical caseloads, organisational issues, lack of support, personal issues, pre-existing mental health symptoms)
- Explore reasons for early retirement/sickness (if relevant)

Managing stress

- Explore how GPs manage their workload/stress in their day-to-day work life, what they do to relax, to look after themselves (self-care strategies: supportive relationships, sport, exercise relaxation techniques)
- Explore relationship with colleagues and whether/how/if concerns are raised, how they are responded
- Explore if GPs have received informal/formal supervision or mentor (1:1 or group) and experience/value of group
- Explore thoughts/feelings about seeking help, barriers to seeking help (stigma/shame, fears about confidentiality, uncertainty of where to go)

For peer review only - http://bmjopen.bmj.com/site/about/guidelines.xhtml

Consolidated criteria for reporting qualitative studies (COREQ): 32-item checklist

Developed from:
Tong A, Sainsbury P, Craig J. Consolidated criteria for reporting qualitative research (COREQ): a 32-item checklist for interviews and focus groups. *International Journal for Quality in Health Care*. 2007. Volume 19, Number 6: pp. 349 – 357

No. Item	Guide questions/description	Reported on Page #
**Domain 1: Research team and reflexivity**		
Personal Characteristics		
1. Interviewer/facilitator	Which author/s conducted the interview or focus group?	Page 5
2. Credentials	What were the researcher's credentials? E.g. PhD, MD	Page 6
3. Occupation	What was their occupation at the time of the study?	Page 1/6
4. Gender	Was the researcher male or female?	Page 1
5. Experience and training	What experience or training did the researcher have?	Page 6
Relationship with participants		
6. Relationship established	Was a relationship established prior to study commencement?	Page 5 .
7. Participant knowledge of the interviewer	What did the participants know about the researcher? e.g. personal goals, reasons for doing the research	Page 5
8. Interviewer characteristics	What characteristics were reported about the inter viewer/facilitator? e.g. Bias, assumptions, reasons and interests in the research topic	Page 6

For peer review only

**Domain 2: study design**		
Theoretical framework		
9. Methodological orientation and Theory	What methodological orientation was stated to underpin the study? e.g. grounded theory, discourse analysis, ethnography, phenomenology, content analysis	Page 6
Participant selection		
10. Sampling	How were participants selected? e.g. purposive, convenience, consecutive, snowball	Page 5
11. Method of approach	How were participants approached? e.g. face-to-face, telephone, mail, email	Page 5
12. Sample size	How many participants were in the study?	Page 5/6
13. Non-participation	How many people refused to participate or dropped out? Reasons?	None
Setting		
14. Setting of data collection	Where was the data collected? e.g. home, clinic, workplace	Page 5 .
15. Presence of non-participants	Was anyone else present besides the participants and researchers?	No
16. Description of sample	What are the important characteristics of the sample? e.g. demographic data, date	Page 5 &15
Data collection		
17. Interview guide	Were questions, prompts, guides provided by the authors? Was it pilot tested?	Page 16
18. Repeat interviews	Were repeat interviews carried out? If yes, how many?	No, due to lack of resources
19. Audio/visual recording	Did the research use audio or visual recording to collect the data?	Page 6

For peer review only

20. Field notes	Were field notes made during and/or after the interview or focus group?	No
21. Duration	What was the duration of the inter views or focus group?	Page 5
22. Data saturation	Was data saturation discussed?	Yes, page 6
23. Transcripts returned	Were transcripts returned to participants for comment and/or correction?	No, due to lack of resources
**Domain 3: analysis and findings**		
Data analysis		
24. Number of data coders	How many data coders coded the data?	Page 6
25. Description of the coding tree	Did authors provide a description of the coding tree?	No
26. Derivation of themes	Were themes identified in advance or derived from the data?	Page 6
27. Software	What software, if applicable, was used to manage the data?	Page 6
28. Participant checking	Did participants provide feedback on the findings?	No, due to lack of resources
Reporting		
29. Quotations presented	Were participant quotations presented to illustrate the themes/findings? Was each quotation identified? e.g. participant number	Pages 7-10
30. Data and findings consistent	Was there consistency between the data presented and the findings?	Yes
31. Clarity of major themes	Were major themes clearly presented in the findings?	Yes
32. Clarity of minor themes	Is there a description of diverse cases or discussion of minor themes?	Yes

For peer review only

BMJ Open

'Treading water but drowning slowly': What are GPs' experiences of living and working with mental illness and distress in England? A qualitative study

Journal:	*BMJ Open*
Manuscript ID	bmjopen-2017-018620.R2
Article Type:	Research
Date Submitted by the Author:	29-Nov-2017
Complete List of Authors:	Riley, Ruth; University of Birmingham, Institute of Applied Health Research; University of Bristol, Social and Community Medicine<br>Spiers, Johanna ; University of Bristol School of Social and Community Medicine<br>Chew-Graham, Carolyn; University of Keele, Primary Care and Health Sciences<br>Taylor, Anna ; University of Bristol, Social and Community Medicine<br>Thornton, Gail; University of Bristol, Social and Community Medicine<br>Buszewicz, Marta; University College London, Research Department of Primary Care and Population Health
**Primary Subject Heading**:	General practice / Family practice
Secondary Subject Heading:	Mental health, Qualitative research, Occupational and environmental medicine
Keywords:	General Practitioners, Depression, Anxiety, Distress, Suicidal Ideation, Chronic stress

SCHOLARONE™
Manuscripts

'Treading water but drowning slowly': What are GPs' experiences of living and working with mental illness and distress in England? A qualitative study

Ruth Riley

Lecturer

Institute of Applied Health Research

University of Birmingham

Birmingham

United Kingdom

r.riley@bham.ac.uk

0121 414 8549

Johanna Spiers

Senior Research Associate

Centre for Academic Primary Care

University of Bristol

United Kingdom

0117 33 14532

Js16447@bristol.ac.uk

Carolyn A. Chew-Graham

Professor of General Practice Research

Research Institute, Primary Care and Health Sciences

Keele University

Keele

United Kingdom

01782 734 717

c.a.chew-graham@keele.ac.uk

For peer review only

Anna Kathryn Taylor

Medical Student

Faculty of Health Sciences

University of Bristol

Bristol

United Kingdom

0117 331 6825

at0012@my.bristol.ac.uk

Gail A. Thornton

Patient and Public Contributor

Centre for Academic Primary Care

University of Bristol

United Kingdom

0117 9104917

gail@thorntonuk.com

Marta Buszewicz

Reader in Primary Care

Research Department of Primary Care and Population Health

University College London

London

United Kingdom

020 7794 0500 extn. 31016

m.buszewicz@ucl.ac.uk

Word count: 3,596

Abstract

Objectives. This paper provides an in-depth account of General Practitioners' (GPs) experiences of living and working with mental illness and distress, as part of a wider study

For peer review only - http://bmjopen.bmj.com/site/about/guidelines.xhtml

reporting the barriers and facilitators to help-seeking for mental illness and burnout, and sources of stress/distress for GP participants.

Design. Qualitative study using in-depth interviews with 47 GP participants. The interviews were audio-recorded, transcribed, anonymised and imported into NVivo11 to facilitate data management. Data were analysed using a thematic analysis employing the constant comparative method.

Setting. England

Participants. A purposive sample of GP participants who self-identified as: 1) currently living with mental distress, 2) returning to work following treatment, 3) off sick or retired early as a result of mental distress or 4) without experience of mental distress. Interviews were conducted face to face or over the telephone.

Results. The findings report GP participants' in-depth experiences of distress and mental illness with many recollecting their distressing experiences and significant psychological and physical symptoms relating to chronic stress, anxiety, depression and/or burnout, and a quarter articulating thoughts of suicide. Many talked about their shame, humiliation and embarrassment at their perceived inability to cope with the stresses of their job and/or their symptoms of mental illness.

Conclusions. These findings paint a concerning picture of the situation affecting primary care doctors, with participants' accounts suggesting there is a considerable degree of mental ill health and reduced wellbeing amongst GPs. The solutions are complex and lie in prevention and provision. There needs to be greater recognition of the components and cumulative effect of occupational stressors for doctors, such as the increasing workload and the clinical and emotional demands of the job, as well as the need for a culture shift within medicine to more supportive and compassionate work environments.

Keywords: Qualitative, General Practitioners, Depression, Anxiety, Distress

For peer review only

Strengths and limitations of this study

- Few studies employing qualitative methods, using in-depth interviews, have been utilised to examine this topic and depict the experience of distress amongst GPs. This study carried out 47 interviews with GPs from across England and contributes to a growing body of research illuminating and examining the experience of living with chronic stress and distress amongst this population of doctors

- Participants were self-selecting, which may be perceived as a limitation; however, the recruitment of participants ensured that the sample was varied in terms of age, gender, number of years as practising GPs, level of seniority/employment status in

For peer review only - http://bmjopen.bmj.com/site/about/guidelines.xhtml

the practice, and geographical location. This variation provided a range of perspectives; however, uniform differences between the groups were not observed.

- The researchers did not employ respondent validation; however, the second coders included academic GPs and team members with lived experience of mental illness which afforded some checks and balances to the validity of the analytic process, interpretation of data and generalizability of the research findings.

- In recruiting individuals with experience of mental illness and burnout, we also included the perspectives of GPs who had no disclosed history of mental illness, which enables the data to be more widely transferable to the UK GP population

- The multi-disciplinary research team independently analysed a sub-set of transcripts in order to contribute to the generation and refinement of codes to maximise rigour. Emergent themes were subsequently discussed by the whole team to ensure credibility and confirmability.

INTRODUCTION

Compared with the general population, doctors, including general practitioners (GPs) working in primary care, experience high levels of psychological distress linked to chronic stress, anxiety, depression and burnout [1-4]. These doctors also have higher rates of mental illness and suicide [5-8]. Evidence from industrialized countries (e.g. UK, USA, Australia), indicates that suicide rates are higher amongst GPs, psychiatrists and anaesthetists compared with other medical specialities [6 8]. Doctors undergoing fitness for practice investigations are at particularly high risk of suicide [9]. Compared with the general population, doctors also have higher rates of drug and alcohol misuse and dependency, with a higher rate of misuse of prescription medicines [10]. Psychological difficulties or distress experienced by doctors can be further compounded by self and societal stigmatising associated with mental illness, with evidence that doctors avoid or delay disclosure and help-seeking due to concerns about loss of confidentiality, denial of illness and logistical barriers to accessing support [11-15]. The prevailing hegemonic culture amongst medical practitioners and non-disclosure of vulnerability, with shame attached to mental health problems or psychological difficulties and the consequent perceived inability to cope within the job, also hinders help-seeking [4 11 16 17]. It has been suggested that personality characteristics such as perfectionism and obsessionality, whilst being positively associated with hard-working and conscientious professionals, may play a role in predisposing doctors towards chronic stress and/or mental ill-health [4].

The increasing prevalence of mental ill-health amongst the medical profession, including GPs, has been attributed to a number of factors, most notably the escalating occupational stresses related to workload, long hours and the emotional demands of the job [4 18-23]. In

For peer review only - http://bmjopen.bmj.com/site/about/guidelines.xhtml

addition, sharp increases in patient demand [19 24] and transfer of care from specialist to primary care [25], as well as reductions in resources [25], have resulted in the pressure on primary care in England being at its highest ever. This pressure is predicted to increase in the future [25]. Findings such as these highlight the potentially stressful context in which GPs are currently working, leaving many vulnerable to distress and/or mental health challenges [26 13 14 23].

Evidence of the extent of mental ill-health and burnout amongst GPs is largely derived from survey data, with some small-scale qualitative studies exploring the topic [13 27-31]. To date, few studies have explored the experiences and accounts of GPs living and working with enduring levels of stress, mental ill-health and psychological distress using in-depth qualitative methods. The employment of in-depth interviews in this study allowed GP participants to describe the full extent and complexity of their distress. In addition, by providing the 'patient's view' [32], qualitative findings can fully elucidate details and context about phenomena in a way that quantitative work cannot [33]. This paper reports GPs' detailed experiences of living and working with mental illness and emotional distress, as part of a wider study reporting the sources of stress and distress[23] for GPs and the barriers and facilitators to help-seeking for mental illness and burnout [14] amongst this medical population.

METHODS

This multi-centre, qualitative study employed in-depth interviews with 47 GPs in England. Information about the study was advertised through professional publications such as Pulse, study newsletters, social media and national and local GP networks (including Local Medical Committees or LMCs) in Bristol, Manchester and London. A sub-sample (n=12) was recruited through a specialist treatment service. GPs interested in taking part were asked to self-select into the following groups: 1: Living with anxiety, depression, stress and/or, burnout; 2: Returning to work following treatment; 3: Off sick or retired early due to psychological ill-health 4: No mental ill-health. GPs were offered reimbursement of £80 to recognise the time for their participation.

GPs who expressed an interest in participating were purposively sampled to represent as even a spread as possible across these four groups, although the largest number of participants were in Group 1. We intended to recruit approximately 10 participants per group. However, the majority of GPs who contacted us self-selected into group one and, due to the emergent rich data, continued recruitment to this group and further exploration of emerging themes was justified in meeting the study's aims and objectives. We endeavored to recruit more participants into groups two and four using targeted publicity information, but because of time constraints those groups remained marginally under-recruited. In the event, many GPs who identified as living with no stress reported as having had experiences of stress and distress at some juncture in their career. More female GPs contacted the study team expressing an interest in participating, and therefore the disparity in numbers interviewed reflects this. The iterative process of recruitment, sampling and analysis ensured that emerging concepts and themes could be tested out amongst participants with different characteristics (e.g. partners vs locum GPs).

For peer review only - http://bmjopen.bmj.com/site/about/guidelines.xhtml

Face-to-face or telephone interviews lasting between 27 and 126 minutes (mean = 69 minutes) were conducted between April and September 2016. The majority of interviews were conducted in participants' homes. The in-depth interviews were conducted by two authors (JS, RR) using a flexible topic guide (see Box 1). This was informed by the existing literature, input from GPs on the study team and PPI consultation exercises conducted with GP networks prior to obtaining funding. The interviews were audio-recorded, transcribed, anonymised and imported into NVivo11 to facilitate data management. Analysis and data collection were conducted iteratively. A thematic analysis was performed involving a process of constant comparison between cases [34]. Analysis commenced with JS generating an initial coding framework grounded in the data, which was added to and refined, with material regrouped and recoded as new data were gathered. Codes were gradually built into broader categories through comparison across transcripts, and higher-level recurring themes were developed until data saturation was reached when no new themes were arising from the data[35]. Reflexivity was employed throughout the research. Both interviewers were experienced qualitative researchers who both reflected on and discussed the impact of the data on their cognitive and emotional sensing throughout the study. Both researchers also discussed and made explicit how their epistemological (JS with a background in psychology and RR in medical sociology) and experiential backgrounds may have oriented the data collection and analytic process.

Members of the multi-disciplinary research team independently analysed a sub-set of transcripts in order to contribute to the generation and refinement of codes to maximise rigour. Emergent themes were discussed by the whole team to ensure credibility and confirmability.

RESULTS

Willing participants were purposively sampled from GPs who expressed an interest in the study, based on demographic and practice characteristics as well as self-selected group type. The characteristics of the participants are found in Table 1.

<<<<insert table 1 here>>>>>

For this paper we will present the following five main themes and corresponding sub themes relating to:

1. Burnout - loss of empathy or compassion fatigue, feeling empty and/or exhausted, difficulty making decisions, low job satisfaction and loss of enjoyment in seeing patients.
2. Anxiety - psychological symptoms relating to anxiety, including feeling anxious or on edge, worrying, irritability, inability to relax and physical symptoms related to anxiety such as panic attacks, hyperventilating, palpitations, sweating and nausea.
3. Depression – distressed at work, insomnia and exhaustion, negative thoughts, low self-esteem, irritability and anger.

For peer review only - http://bmjopen.bmj.com/site/about/guidelines.xhtml

4. Suicidal ideation - thoughts of suicide.
5. Shame, as well as feelings of failure and humiliation, resulting from a perceived lack of resilience and inability to cope.

It should be noted that some participants described or disclosed symptoms that could be clearly attributed to chronic stress, anxiety, depression and burnout. Other participants described or framed their symptoms in terms of diagnostic concepts associated with these categories, whilst others talked about their feelings and emotional state without necessarily labelling them. Notably, the symptomology was identified across all groups including Group 4, that is individuals who self-identified as having no mental health concerns. The themes also applied to participants irrespective of seniority or role in the practice, age or gender. There was no clear distinction in terms of presentation or severity of symptoms between the groups and so no typology could be developed along these lines.

1. Symptoms of Burnout

Some participants reported feeling a loss of empathy or compassion fatigue, feeling empty, exhaustion, difficulty making decisions, low job satisfaction and loss of enjoyment for their work, using language which highlights the depth of the feelings:

I'd lost my empathy with my patients. You know, and er I'm just – I was just – you're just like a ketchup bottle in a production line […] But from a personal point of view I'm still feeling a bit washed out by it, you know, I feel a little bit empty. You know, I feel like I'm burnt out really a bit, to be honest. (P25, Male Salaried)

I was saying all the right things but […] I felt quite detached from it. (P27, Female Salaried)

The following participant describes how feeling overworked can lead to a reduced ability to offer compassion and support to patients:

If you find yourself working too many sessions, I can, you know, I feel it, and you almost feel like you lose the milk of human kindness. And you can't do that job if you're not (pause) at your best. (P21, Female, Salaried and Locum)

2. Symptoms of Anxiety

The majority of participants, including those who self-identified as having no mental health concerns (Group 4), described psychological symptoms relating to anxiety, including feeling on edge or hyper-alert, persistent worrying, irritability and an inability to relax. The following examples demonstrate how difficult and distressing these symptoms and feelings were for some participants to negotiate:

For peer review only - http://bmjopen.bmj.com/site/about/guidelines.xhtml

Everything was running around in my mind at 200 miles an hour. I was in sort of hyper drive and, yeah, you wake up to super stimulated, super awake, super, you know, on edge and anxious. (P36, Male Partner)

Many participants also described physical symptoms related to their anxiety, such as panic attacks, hyperventilating, palpitations, sweating, and nausea:

Erm (pause) and I, and I was getting quite bad chest pain, palpitations, I was feeling anxious in many circumstances, like the school run, just meeting people, going to meetings and it was becoming incapacitating where it felt like somebody had got a knife in my chest all the time. And I knew it wasn't anything to do with my heart. (P31, Female Locum)

3. Symptoms of Depression

Many GP participants described symptoms relating to depression, which included: feeling distressed at work, low mood, hopelessness, sleep problems, low self-esteem, guilt, restlessness, fatigue and suicidal feelings. Some participants employed a range of poignant and powerful metaphors which illustrate the extent of those difficult emotions:

And, to be honest with you, that kind of er – the best way that I could describe that is that it's kind of like a dark shadow that's in the corner of the room […] I basically felt like I was treading water but drowning slowly. (P45, Male Salaried)

Distressed at work:

Many participants recalled their experiences of feeling distressed, anxious and tearful, with some describing breaking down at home, after or during work or in front of patients:

I mean there were times certainly when I arrived at work and just sat outside and cried before I even went in the building. And I often cried on the way home. I'd often cry when I came in from work, um just collapse in a heap, and get to bed actually crying. (P31, Female Locum)

I remember once actually sitting under the desk, hugging my knees to my chest, I was so anxious. (P25, Male Salaried)

I would just end up in tears all the time at the end of each session. (P53, Female Salaried)

Insomnia and exhaustion:

Amongst the myriad of symptoms, insomnia and exhaustion were a commonplace experience for study participants:

Um - so I wasn't sleeping and I wasn't - wasn't eating very well, and - and it just all sort of took its toll. (P23, Female Salaried)

For peer review only - http://bmjopen.bmj.com/site/about/guidelines.xhtml

It's very hard to function when you're not ever getting refreshing sleep […] And I just wouldn't stop. And absolute, incapacitating terror. Um - So, but one's having um — night, night terrors maybe two, three, four times a week. Sometimes more than one a night. (P24, Female Partner)

Many GP participants detailed their exhaustion, which was often linked to insomnia and could be symptomatic of their anxiety, depression and/or feeling burnt out, demonstrating the ways in which different elements of GP experience combine to add to distress:

Probably like sort of swimming through treacle, so everything seemed - um - much harder. (P38, Male Partner)

So like you can't, you can't avoid that fatigue. You might be able to put it off a bit (laughs) but you're going to pay interest on it. (P7, Female Locum)

Negative thoughts/low self-esteem:

Some participants talked about grappling with cycles of negative thoughts, symptomatic of low self-esteem and a common symptom of depression, as this participant illustrates:

And so then I'm really struggling, because I'm struggling with my internal dialogue saying, you know, "Not good enough, could be doing a better job. Not really, not really er making people better." […] persistent negative cycles of er (pause), yeah, of negative thoughts about yourself (P20, Male Partner)

Irritability and anger:

Some study participants described feeling irritable and sometimes angry; a possible symptom of depression in this context:

But I think, towards the end when I stopped locum-ing again, I was beginning to feel very tired as a result and that it was taking more out of me than it ought to really, and feeling um, yeah, feeling more irritable. (P47, Female on sick leave)

This account demonstrates how feeling stressed and irritable can sometimes manifest itself within a work context:

…it was fairly um traumatic really, I have to say…. I think I'd become quite stressed at work actually and I'd lost my temper in meetings at times and um nobody quite knew what to do about it. (P39, Male Partner)

4. Suicidal ideation

For peer review only - http://bmjopen.bmj.com/site/about/guidelines.xhtml

One in four GP participants described having had suicidal thoughts, with the following participants reflecting on occasions when they had contemplated and ruminated on ways of taking their own lives:

And it just came on me like a wave. And then I started thinking, "I'm going to jump onto those train tracks." (P45, Male Salaried)

And um constant ruminations on suicide and doing myself in. And the trick with suicide is to persuade yourself that those around you would be better off without you, um and I got to that point and I decided on ways that I might (pause) do myself in and ruminated about that all the time. Just, it just filled my waking and sleeping nights. (P20, Male Partner)

This latter data extract demonstrates how suicidal thoughts can be both constant and normalised for some GPs.

The following GP reflects on the advice she gives to her patients who express suicidal thoughts and how her feelings mirrored her patients' expressed hopelessness and despair, again showing the ways in which GP experiences can combine to add to distress:

Um, and then I think the hard thing, the worst thing I think I found was when people were feeling suicidal, and they were saying they were feeling suicidal and why they were feeling suicidal. And normally I'd be like, "Look, you know, it's temporary, it'll get better. These are the things that we can do," but I just sat there thinking, "Well, you're probably right, it is rubbish," you know. (P27, Female Partner)

5. Shame and feelings of failure

Perhaps symptomatic of the stigma attached to mental illness and the culture of invulnerability within medicine, some GP participants described how they felt ashamed, embarrassed, humiliated and a sense of having failed, due to their perceived lack of resilience and inability to cope. These quotations illustrate the depth of that emotion for two of the participants:

And you do feel a huge sense of shame, huge sense of shame. Because even though I treat patients day in, day out with anxiety and depression, it felt like this was my not coping. And I was thinking, "Why are the partners all able to cope with this but not me?" and that sort of thing. So it was all rolled into one really. (P4, Female Partner)

Er (sigh) yeah, I think for me it was very embarrassing. Didn't want to admit it. Couldn't believe that like I'd got to this stage. Cos I'd never been someone like that […] yeah, I did find it really hard, I think, to actually accept that this had happened. Um (pause) yeah, bit of a failure? I suppose, in a way. Kind of feel like you're not strong enough? (P44, Female Registrar)

The participant below highlights how the denial and intolerance of mental ill health amongst some colleagues can enter into an individual's internal dialogue, thus compounding existing feelings of shame:

For peer review only - http://bmjopen.bmj.com/site/about/guidelines.xhtml
For peer review only

'Yeah, well you don't get ill, do you? You know, you, you refuse to accept any illness in yourself, don't you? It's just a question of that's why doctors present late with illnesses because they don't like to make a fuss. We see it as a sign of weakness, which I think probably is why we are intolerant of illness in our partners, because we're doctors. And I think it's just part of the sort of macho thing which is first drummed into you at medical school and the end product of that generation, which – if you can't take the heat get out of the kitchen, type thing'. (P49, Female, Retired)

DISCUSSION

The accounts of the 47 participants in this study suggest there may be an undetermined population of GPs who are living and working with poor mental health. Participants vividly described and recollected their distressing experiences and troubling symptoms, with one in four GPs articulating thoughts of suicide. Such findings are currently underreported in the literature with few studies providing evidence of suicidal thoughts and extreme levels of distress amongst this GP population. The GP participants described numerous troubling psychological and physical symptoms relating to chronic stress, anxiety, depression and/or burnout with several breaking down at work – crying, feeling despair and some contemplating suicide. Many also talked about their shame, humiliation and embarrassment at their perceived inability to cope with the stresses of their job and/or their symptoms of mental ill-health. The experiences and feelings of many GP participants depict a dispiriting emotional landscape, with notable signs of poor mental health and striking levels of distress.

There has been a paucity of in-depth qualitative research exploring this topic area with doctors, including GPs [14]. This study highlights that many GPs are living *and working* with extreme levels of emotional distress. A notable proportion of participants vividly recalled and articulated both fleeting and recurring thoughts of suicide, which is reflective of the extent of individuals' distress and suffering. These candid, evocative and sometimes disturbing accounts, some hitherto unvoiced, reveal a population of doctors with generally very poor mental health.

The findings from this study and the related papers[14 23] were based on in-depth interviews with 47 GPs from across England and contribute to a growing corpus of research illuminating and examining the causes, experiences and impact of chronic stress and distress amongst GPs [20 36]. The participants were self-selecting, which may be seen as a limitation; however, the recruitment of participants ensured that the sample was varied in terms of age, gender, number of years as practising GPs, level of seniority/employment status in the practice, and geographical location. In addition to recruiting individuals with self-reported mental ill-health and burnout, we also included the perspectives of GPs who had no disclosed history of mental health problems, which enables the data to be more transferable to the wider GP population. However, despite this heterogeneity within the sample, we did not observe any marked differences between the groups, suggesting a certain uniformity of distress for the group despite demographic differences.

For peer review only - http://bmjopen.bmj.com/site/about/guidelines.xhtml

These findings present a concerning picture of psychological difficulties and reduced wellbeing affecting primary care doctors. The experience of chronic stress and anxiety in GPs (e.g. feeling tense, restless, anxious, worried or having disrupted sleep, panic attacks, hyperventilating, palpitations, sweating, and nausea) appears commonplace, including in those who did not identify themselves as having mental ill-health. Our findings reflect the evidence that doctors frequently normalise, minimise, deflect or downplay how stressed they feel, which may preclude help-seeking and the availability of appropriate support [37]. Strikingly, one GP participant normalised the way they routinely ruminated on their suicidal thoughts, with other participants indicating that they were living and coping on a day-to-day basis with symptoms of chronic stress, anxiety and depression, ranging from sleeplessness to persistent anxiety. Evidence indicates that chronic stress negatively impacts on physical health and is a key risk factor for mental illness, including anxiety and depression, and can lead to the adoption of maladaptive coping mechanisms such as nicotine, alcohol, and illicit drug use [38].

Normalising and acknowledging chronic stress as an occupational hazard associated with being a doctor is helpful, but only if solutions are sought which focus on prevention and the provision of support to maintain the health and wellbeing of frontline clinicians. Since January 2017, GPs and GP trainees in England suffering from mental ill health and addiction have been able to access free support through the National Health Service's GP Health Service [39]. Further research and evaluation must be undertaken to assess the effectiveness and accessibility of this service. The emphasis now needs to focus on preventing mental ill health and improving the support available to doctors. This findings highlight that internalised and perceived stigma still prevent doctors from seeking support[11].

Access to personal, relational and environmental resources are protective factors for good mental health and wellbeing and impact on individuals' resilience [40]. Crucially, collegial support is a protective factor for good mental health - support from mentors, supervisors and colleagues is associated with resilience and reduced sickness [22].

Balint groups or similarly structured group work or supervision continue to be employed in general practice and are valued by GPs [41], yet are not used across all practices. Individual or group supervision aims to provide a safe and supportive space where staff can openly discuss the pressures and emotional challenges of their work and may, as previous evidence suggests, provide GPs with the support they need whilst offering protection against compassion fatigue and burnout [42]. Its wider use may need to be considered.

Tackling the culture of invulnerability early on in medical training is also key. Schwartz Centre Rounds®, for instance, are currently being piloted in medical schools with early evidence supporting their value [43]. First5®[44], buddy systems, and access to regular supervision or mentorship can also offer support and a reflective space. The British Medical Association (BMA) have produced guidance on recognising signs of mental distress amongst colleagues with suggestions about what action to take [45]. Perhaps caution needs to be applied to the over-reliance on personal resilience as a solution as this may shift the responsibility for maintaining good mental health entirely on to the individual. While self-care is paramount in maintaining good mental and physical health, it is not solely the

For peer review only - http://bmjopen.bmj.com/site/about/guidelines.xhtml

responsibility of individual doctors to look after their mental health; employers and colleagues need to engender a more supportive and compassionate culture. Doctors are not invulnerable to suffering, most especially their own.

In the light of the findings of this study, we recommend the following:

- Greater recognition of the components and cumulative effect of the occupational stressors for doctors, such as increasing workload and the clinical and emotional demands of the job.
- A culture change within medicine, with a shift to more supportive and compassionate work cultures.
- The de-stigmatising of mental ill-health and the recognition of GPs' experiences and emotional responses as symptomatic of occupational stress as opposed to any perceived failure to cope in the individual.

Conclusion

These findings, together with those from the wider study[14 23], paint a concerning picture of the situation affecting primary care doctors, with participants' accounts suggesting there is a population of GPs experiencing a considerable degree of mental ill health, and overall reduced wellbeing amongst GPs. The solutions are complex and prevention lies in addressing the systemic causes of stress (related to diminishing resources, increased workload, etc.). In addition to recognising the components and cumulative effect of occupational stressors for doctors, such as the increasing workload and the clinical and emotional demands of the job, there is need for a culture shift within medicine to more supportive and compassionate work environments where it is safe and acceptable for doctors to talk or seek help when they need it Medical schools, medical institutions and individuals need to address and tackle the stigma of mental illness and engender a greater tolerance and acceptance of invulnerability

Author Contribution:

RR, JS, CCG, MB– substantial contributions to conception and design, acquisition of data, or analysis and interpretation of data; drafting the article or revising it critically for important intellectual content; and final approval of the version to be published.

AT & GT - analysis and interpretation of data; drafting the article or revising it critically for important intellectual content; and final approval of the version to be published.

Acknowledgements: The authors would like to thank all volunteer participants who kindly gave their time to share their experiences of working in English general practice; and collaborators Dr Clare Gerada and Dr Chris Manning for endorsing this study, providing valuable comments on the funding application and assisting with recruitment.

Competing interests: None

For peer review only - http://bmjopen.bmj.com/site/about/guidelines.xhtml

Funding: The study was funded by NIHR School for Primary Care Research

Department of Health Disclaimer: The views and opinions expressed therein are those of the authors and do not necessarily reflect those of the NIHR School for Primary Care Research, NIHR, NHS or the Department of Health.

Ethical approval: Ethical approval was granted by the South West - Frenchay Research Ethics Committee (reference number: 15/SW/0350)

Data sharing statement: This study has not received ethical approval to share confidential data with any third party other than the study research team.

For peer review only

REFERENCES

1. Chopra SS, Sotile WM, Sotile MO. Physician burnout. JAMA 2004;**291**(5):633-33.
2. Merlo LJ, Singhakant, S., Cummings S.M., and Cottler L.B. Reasons for Misuse of Prescription Medication Among Physicians Undergoing Monitoring by a Physician Health Program. Journal of addiction medicine 2013;**7**(5):349-53.
3. Orton P, Orton C, Pereira Gray D. Depersonalised doctors: a cross-sectional study of 564 doctors, 760 consultations and 1876 patient reports in UK general practice. BMJ Open 2012;**2**(1).
4. Brooks SK, Gerada, C., Chalder, M. Review of literature on the mental health of doctors: Are specialist services needed? Journal of Mental Health 2011:1-11.
5. Dobbin A. Burnt out or fired up? Helping recovery in distressed patients can increase our own resilience. Br J Gen Pract 2014;**64**(627):497-98.
6. Hawton K, Clements A, Sakarovitch C, et al. Suicide in doctors: a study of risk according to gender, seniority and specialty in medical practitioners in England and Wales, 1979–1995. Journal of Epidemiology and Community Health 2001;**55**(5):296-300.
7. Richings JC, Khara, G. S. & Mcdowell, M. Suicide in young doctors. British Journal of Psychiatry 1986;**149**:475-78.
8. Schernhammer ES, Colditz GA. Suicide Rates Among Physicians: A Quantitative and Gender Assessment (Meta-Analysis). American Journal of Psychiatry 2004;**161**(12):2295-302.
9. GMC. Doctors who commit suicide while under GMC fitness to practise investigation. 2014.
10. Brooks SK, Chalder T, Gerada C. Doctors vulnerable to psychological distress and addictions: treatment from the Practitioner Health Programme. J Ment Health 2011;**20**:157–64.
11. Henderson M, Brooks SK, del Busso L, et al. Shame! Self-stigmatisation as an obstacle to sick doctors returning to work: a qualitative study. BMJ Open 2012;**2**(5).
12. Cohen D, Winstanley SJ, Greene G. Understanding doctors' attitudes towards self-disclosure of mental ill health. Occupational Medicine 2016;**66**(5):383-89.
13. Thompson WT, Cupples ME, Sibbett CH, et al. Challenge of culture, conscience, and contract to general practitioners' care of their own health: qualitative study. British Medical Journal 2001;**323**:728–31.
14. Spiers J, Buszewicz M, Chew-Graham CA, et al. Barriers, facilitators, and survival strategies for GPs seeking treatment for distress: a qualitative study. Br J Gen Pract 2017.
15. Cheshire A, Hughes J, Lewith G, et al. GPs' perceptions of resilience training: a qualitative study. Br J Gen Pract.
16. Department of Health. Mental health and ill health in doctors London: Department of Health, 2008.
17. Gold KJ, Andrew LB, Goldman EB, et al. "I would never want to have a mental health diagnosis on my record": A survey of female physicians on mental health diagnosis, treatment, and reporting. General hospital psychiatry 2016;**43**:51-57.

For peer review only - http://bmjopen.bmj.com/site/about/guidelines.xhtml

18. Baird B, Charles A, Honeyman M, et al. Understanding pressures in general practice. 2016.
19. Hobbs FR, Bankhead C, Mukhtar T, et al. Clinical workload in UK primary care: a retrospective analysis of 100 million consultations in England, 2007–14. The Lancet 2016.
20. Doran N, Fox F, Rodham K, et al. Lost to the NHS: a mixed methods study of why GPs leave practice early in England. Br J Gen Pract 2016;**bjgpfeb**.
21. Firth-Cozens J. A perspective on stress and depression. In: J. Cox JK, A. Hutchinson & P. McAvoy, ed. Understanding doctors' performance. Oxford: Radcliffe Publishing., 2006:22–25.
22. Firth-Cozens JH, J. *A Little too Much, in How to Survive in Medicine: Personally and professionally*. Oxford: Wiley-Blackwell, 2010.
23. Riley R, Spiers J, Buszewicz M, et al. 'What are the Sources of Stress and Distress for General Practitioners Working in England? A Qualitative Study' BMJ Open 2017.
24. Hippisley-Cox J, Vinogradova Y. Trends in Consultation Rates in General Practice 1995 to 2008: Analysis of the QResearch database. London: NHS Information Centre, 2009.
25. King's Fund. Improving the quality in General Practice. London: King's Fund, 2011.
26. Brooks SK, Gerada C, Chalder M. Review of literature on the mental health of doctors: Are specialist services needed? Journal of Mental Health 2011:1-11.
27. Ahola K, Hakanen J. Job strain, burnout, and depressive symptoms: A prospective study among dentists. J Affect Disorders 2007;**104**:103 - 10.
28. Clarke J, O'Sullivan Y, Maguire N. A study of self-care among Irish doctors. Irish medical journal 1997;**91**(5):175-76.
29. DoH. National Survey of General Practitioner Job Satisfaction. 2012.
30. Dyrbye LN, Varkey P, Boone SL, et al. Physician satisfaction and burnout at different career stages. Mayo Clin Proc 2013;**88**(12):1358-67.
31. Firth-Cozens J. Predicting stress in general practitioners: 10 year follow up postal survey. British Medical Journal of Addiction Medicine 1997;**315**:34–35.
32. Armstrong D. The patient's view. Social science & medicine 1984;**18**(9).
33. Smith JA. *Qualitative psychology: A practical guide to research methods*: Sage., 2007.
34. Fram SM. The constant comparative analysis method outside of grounded theory. The Qualitative Report 2013;**18**(1):1.
35. Sandewaloski MS. Sample size in qualitative research. Res Nurs Health 1995;**18**:179-83.
36. Fisher RFR, Croxson CHD, Ashdown HF, et al. GP views on strategies to cope with increasing workload: a qualitative interview study. British Journal of General Practice 2017.
37. Thompson N, Corbett, SS & Welfare, M. A qualitative study of how doctors use impression management when they talk about stress in the UK. International Journal of Medical Education 2013;**4**:236-46.
38. L W, Hange D, Björkelund C, et al. Prevalence of perceived stress and associations to symptoms of exhaustion, depression and anxiety in a working age population seeking primary care-an observational study. BMC Fam Pract 2015;**16**(38).
39. NHS England. NHS GP Health Service. Secondary NHS GP Health Service 2017. https://www.england.nhs.uk/gp/gpfv/workforce/health-service/.
40. Cusack L, Smith M, Hegney D, et al. Exploring environmental factors in nursing workplaces that promote psychological resilience: constructing a unified theoretical model? Front Psychol 2016;**7**:1-8.
41. Van Roy K, Vanheule S, R I. Research on Balint groups: a literature review. Patient Educ Couns 2015;**98**(6):685–94.
42. Benson J, Magraith K. Compassion fatigue and burnout: the role of Balint groups. Australian family physician 2005;**34**(6):497-8.
43. Gishen F, Gill, D. Introducing Schwartz rounds at UCL Medical School. Secondary Introducing Schwartz rounds at UCL Medical School.

For peer review only - http://bmjopen.bmj.com/site/about/guidelines.xhtml

https://uclmsnews.wordpress.com/2014/09/29/introducing-schwartz-rounds-at-ucl-medical-school/. 15/09/2017

44. RCGP. http://www.rcgp.org.uk/membership/about-rcgp-membership/join-rcgp-newly-qualified-gps.aspx. Accessed 24/11/2017

45. BMA. Are you concerned about a colleague? Secondary Are you concerned about a colleague?. https://www.bma.org.uk/advice/work-life-support/your-wellbeing/concerned-about-a-colleague. Accessed 24/11/2017

Table 1: Participant and practice characteristics

**GP Characteristics**	**N=47**
	N
Sex (female)	33
Age	
20-29	1
30-39	12
40-49	20
50-59	14
Group	
1	19
2	9
3	11
4	8
Years since qualified	
<10	19
No of sessions contracted per week	
<5 sessions (mean actual hours worked)	12 (15)
>5 sessions (mean actual hours worked)	32 (38)
Fully retired	3
Mean size of practice	12624
Range	3,600 – 37,000
Employment status	
Partner	17
Salaried	11
Locum	5
Registrar	4
Retired	3
Sick leave	5
More than one role	2

For peer review only - http://bmjopen.bmj.com/site/about/guidelines.xhtml

For peer review only

Box 1. Interview topic guide

Introduction and background

- Describe average working day in practice (hours, surgery, home-visits) and any additional responsibilities

Current well-being

- Explore current well-being, feelings about work, levels of stress, work-life balance
- Explore causes of stress/distress (work load, hours, admin, clinical caseloads, organisational issues, lack of support, personal issues, pre-existing mental health symptoms)
- Explore reasons for early retirement/sickness (if relevant)

Managing stress

- Explore how GPs manage their workload/stress in their day-to-day work life, what they do to relax, to look after themselves (self-care strategies: supportive relationships, sport, exercise relaxation techniques)
- Explore relationship with colleagues and whether/how/if concerns are raised, how they are responded
- Explore if GPs have received informal/formal supervision or mentor (1:1 or group) and experience/value of group
- Explore thoughts/feelings about seeking help, barriers to seeking help (stigma/shame, fears about confidentiality, uncertainty of where to go)

For peer review only

For peer review only - http://bmjopen.bmj.com/site/about/guidelines.xhtml
